# Metabolism and Occurrence of Methanogenic and Sulfate-Reducing Syntrophic Acetate Oxidizing Communities in Haloalkaline Environments

**DOI:** 10.3389/fmicb.2018.03039

**Published:** 2018-12-10

**Authors:** Peer H. A. Timmers, Charlotte D. Vavourakis, Robbert Kleerebezem, Jaap S. Sinninghe Damsté, Gerard Muyzer, Alfons J. M. Stams, Dimity Y. Sorokin, Caroline M. Plugge

**Affiliations:** ^1^Laboratory of Microbiology, Wageningen University & Research, Wageningen, Netherlands; ^2^European Centre of Excellence for Sustainable Water Technology, Wetsus, Leeuwarden, Netherlands; ^3^Microbial Systems Ecology, Department of Freshwater and Marine Ecology, Institute for Biodiversity and Ecosystem Dynamics, University of Amsterdam, Amsterdam, Netherlands; ^4^Department of Biotechnology, Delft University of Technology, Delft, Netherlands; ^5^Department of Marine Microbiology and Biogeochemistry, NIOZ Netherlands Institute for Sea Research, Utrecht University, Utrecht, Netherlands; ^6^Department of Earth Sciences, Faculty of Geosciences, Utrecht University, Utrecht, Netherlands; ^7^Centre of Biological Engineering, University of Minho, Braga, Portugal; ^8^Winogradsky Institute of Microbiology, Research Centre of Biotechnology, Russian Academy of Sciences, Moscow, Russia

**Keywords:** syntrophic acetate oxidation, haloalkaliphiles, soda lakes, syntrophy, SAOB, syntrophic acetate oxidizing bacteria, genome-centric metagenomics

## Abstract

Anaerobic syntrophic acetate oxidation (SAO) is a thermodynamically unfavorable process involving a syntrophic acetate oxidizing bacterium (SAOB) that forms interspecies electron carriers (IECs). These IECs are consumed by syntrophic partners, typically hydrogenotrophic methanogenic archaea or sulfate reducing bacteria. In this work, the metabolism and occurrence of SAOB at extremely haloalkaline conditions were investigated, using highly enriched methanogenic (M-SAO) and sulfate-reducing (S-SAO) cultures from south-western Siberian hypersaline soda lakes. Activity tests with the M-SAO and S-SAO cultures and thermodynamic calculations indicated that H_2_ and formate are important IECs in both SAO cultures. Metagenomic analysis of the M-SAO cultures showed that the dominant SAOB was ‘*Candidatus* Syntrophonatronum acetioxidans,’ and a near-complete draft genome of this SAOB was reconstructed. ‘*Ca.* S. acetioxidans’ has all genes necessary for operating the Wood–Ljungdahl pathway, which is likely employed for acetate oxidation. It also encodes several genes essential to thrive at haloalkaline conditions; including a Na^+^-dependent ATP synthase and marker genes for ‘salt-out‘ strategies for osmotic homeostasis at high soda conditions. Membrane lipid analysis of the M-SAO culture showed the presence of unusual bacterial diether membrane lipids which are presumably beneficial at extreme haloalkaline conditions. To determine the importance of SAO in haloalkaline environments, previously obtained 16S rRNA gene sequencing data and metagenomic data of five different hypersaline soda lake sediment samples were investigated, including the soda lakes where the enrichment cultures originated from. The draft genome of *‘Ca.* S. acetioxidans’ showed highest identity with two metagenome-assembled genomes (MAGs) of putative SAOBs that belonged to the highly abundant and diverse *Syntrophomonadaceae* family present in the soda lake sediments. The 16S rRNA gene amplicon datasets of the soda lake sediments showed a high similarity of reads to ‘*Ca.* S. acetioxidans’ with abundance as high as 1.3% of all reads, whereas aceticlastic methanogens and acetate oxidizing sulfate-reducers were not abundant (≤0.1%) or could not be detected. These combined results indicate that SAO is the primary anaerobic acetate oxidizing pathway at extreme haloalkaline conditions performed by haloalkaliphilic syntrophic consortia.

## Introduction

Syntrophic acetate oxidation (SAO) is an anaerobic process where two microorganisms are responsible for the degradation of acetate. In this process, syntrophic acetate oxidizing bacteria (SAOB) oxidize acetate and produce H_2_ and CO_2_ or formate. Hydrogen and formate can serve as interspecies electron carriers (IECs) that are utilized by syntrophic partners, which in most cases are hydrogenotrophic methanogens or sulfate-reducing bacteria (SRB). Only a few bacterial species able to perform SAO have been described, such as “strain AOR” (Lee and Zinder, 1988), *Clostridium ultunense* (Schnürer et al., 1996), *Thermoacetogenium phaeum* (Hattori et al., 2005), *Tepidanaerobacter acetatoxydans* (Westerholm et al., 2011), *Pseudothermotoga lettingae* (Balk et al., 2002) and *Syntrophaceticus schinkii* (Westerholm et al., 2010). Acetate is an important intermediate in the anaerobic degradation of organic matter and up to 80% of produced methane can derive from acetate (Mountfort and Asher, 1978; Lovley and Klug, 1982). In highly reduced environments, acetate is degraded by aceticlastic methanogens or by sulfate-reducing bacteria (SRB). However, aceticlastic methanogenesis is inhibited at extreme conditions, such as high ammonia, high fatty acid concentrations and high temperatures and as a result, SAO becomes the dominant acetate utilizing process (Shigematsu et al., 2004; Karakashev et al., 2006; Nozhevnikova et al., 2007; Noll et al., 2010; Westerholm et al., 2012). Methanogenic digesters of protein rich organic matter exhibit high concentrations of ammonium, which strongly inhibits aceticlastic methanogenesis (Sprott and Patel, 1986; Steinhaus et al., 2007; Manzoor et al., 2016) and results in SAO to become the dominant acetate degradation pathway (Schnürer et al., 1994, 1999; Schnürer and Nordberg, 2008; Westerholm et al., 2012; Jiang et al., 2018). SAO has also been reported to be an important anaerobic process at high temperature (55°C) oil fields (Mayumi et al., 2011, 2013; Dolfing, 2014) and acetate utilization shifted from aceticlastic methanogenesis to SAO at higher temperatures in rice paddy field soils (Rui et al., 2011).

Extreme conditions also exist in hypersaline soda lakes, which are a specific type of salt lakes characterized by double extremes; high pH (9.5–11) and sodium carbonate/bicarbonate concentrations up to saturation (0.5–4 M Na^+^) (Sorokin et al., 2014b). Recently, SAO communities have been enriched from hypersaline soda lakes and the dominant SAOB, ‘*Candidatus* Syntrophonatronum acetioxidans,’ represents a novel genus within the family *Syntrophomonadaceae* (Sorokin et al., 2014a, 2015a, 2016). The SAO pathways used by these first haloalkaliphilic enrichment cultures, the mechanisms of energy conservation, and the IEC that is transferred between the partner organisms are, however, unknown. In this study, we investigated the occurrence and metabolism of SAO using these enrichment cultures of ‘*Ca.* S. acetioxidans’ with either the hydrogenotrophic methanogenic partner *Methanocalculus natronophilus* strain AMF5 (M-SAO) (Sorokin et al., 2015a, 2016) or the sulfate-reducing partner *Desulfonatronovibrio magnus* (S-SAO) (Sorokin et al., 2011). Acetate oxidation, IEC formation, and rates of methane or sulfide formation, respectively, were monitored in the presence and absence of inhibitors of methanogenesis or sulfate-reduction and in presence of possible IECs (i.e., formate or H_2_). A draft genome of the SAOB ‘*Ca.* S. acetioxidans’ was obtained from the metagenome of the M-SAO enrichment culture to identify the genes putatively involved in acetate-oxidizing pathways and the adaptation mechanisms to haloalkaliphilic conditions. The composition of membrane lipids was investigated for possible adaptations to these conditions. The occurrence and ecology of SAOBs and aceticlastic methanogens and acetate-degrading sulfate reducers was investigated using 16S rRNA gene amplicon and metagenome sequencing datasets from five different soda lake sediment samples of south-western Siberia that were published recently (Vavourakis et al., 2018). The sediment of one of these lakes, Bitter-1, was also the inoculum of the studied SAO enrichment cultures. These results provide more insights into the importance of SAO in these extreme environments.

## Materials and Methods

### Inoculum

The enrichment cultures used were described previously and consist of ‘*Ca.* S. acetioxidans’ (Sorokin et al., 2014a) together with the haloalkaliphilic hydrogenotrophic methanogen *Methanocalculus natronophilus* strain AMF5 (M-SAO) (Sorokin et al., 2015a, 2016) or the hydrogenotrophic sulfate-reducer *Desulfonatronovibrio magnus* (S-SAO) (Sorokin et al., 2011). All cultures derived from Bitter-1 soda lake sediment (south-western Siberia).

### Media and Cultivation

The sodium carbonate-bicarbonate alkaline media (1 M Na^+^ and pH 9.5) used in these experiments was made as described previously (Sorokin et al., 2014a). SAO enrichment cultures and pure cultures of hydrogenotrophic partners were pre-grown in media with either 60 mM acetate or 80 mM formate and 2 mM acetate (all sodium salts), respectively. For methanogenic SAO and pure cultures, 0.1 mM coenzyme M was supplemented. For sulfate-reducing SAO or pure cultures, 20 mM sodium sulfate was added as electron acceptor. All incubations were done at 30°C, as described previously (Sorokin et al., 2014a).

When pre-grown methanogenic SAO cultures had consumed around 30 mM acetate, the gas phase of the cultures was exchanged with 100% (v/v) N_2_. Afterwards, 30 ml of pre-grown cultures was added to autoclaved, crimp-capped, 100% (v/v) N_2_-containing 50 ml serum vials using a nitrogen-flushed syringe. Incubations were done in triplicate with either (1) 45 mM acetate – static incubation, (2) 45 mM acetate – shaking at 130 rpm, (3) 45 mM acetate with 100% (v/v) H_2_, (4) 45 mM acetate and 7 mM or 80 mM formate, (5) 45 mM acetate and 10 mM bromoethanesulfonate (BES).

When pre-grown sulfate-reducing SAO cultures had consumed around 20 mM acetate, the cells were collected by centrifugation (1 h at 4000 × *g*) and resuspended in fresh media followed by gas exchanging with 100% (v/v) N_2_ to remove produced sulfide. Afterwards, 30 ml of pre-grown culture was added to autoclaved, crimp-capped, 100% (v/v) N_2_-containing 50 ml serum vials using a nitrogen-flushed syringe. Incubations were done in triplicate with either (1) acetate and sulfate – static incubation, (2) acetate and sulfate – shaking, (3) acetate and sulfate with 100% (v/v) H_2_, (4) acetate, sulfate and 80 mM formate, and (5) acetate and 5 mM molybdate ($MoO_{4}^{2-}$). Acetate was added after 65 h of incubation to a total concentration of around 25 mM (the concentration of acetate was 5 mM before additional acetate supplementation). Acetate was amended later after it was confirmed that the cultures were still active after washing. There was less acetate amended than to the methanogenic cultures to prevent overproduction of sulfide that is both inhibitory for the cultures and more difficult to measure at high concentrations.

All cultures were incubated statically at 30°C, except for condition 2 that was shaken at 130 rpm. For all cultures, H_2_, organic acids (mainly formate and acetate), sulfate and sulfide were monitored during incubation. Methanogenic cultures were incubated for a total of 286 h while sulfate-reducing cultures were incubated for 584 h.

Additionally, the production of formate from H_2_ and *vice versa* during growth of the syntrophic partners in pure culture was investigated. For this work, pure cultures of *Methanocalculus natronophilus* strain AMF5 and *Desulfonatronovibrio magnus* were pre-grown with 100% (v/v) H_2_. After full growth, cultures were gas exchanged with 100% (v/v) N_2_ and 10% (v/v) of the cultures was transferred to new media with either 100% (v/v) H_2_ or 100 mM formate. Growth of these pure cultures was monitored by measuring optical density at 600 nm and H_2_, formate and methane or sulfide formation over time.

### Analytical Measurements

#### Headspace Gasses

Headspace gas samples (0.2 ml) from the incubations were taken at 20°C using a sterile N_2_-flushed syringe and analyzed using a Compact GC (Global Analyser Solutions, Breda, Netherlands) equipped with a Carboxen 1010 pre-column, followed by two lines: a Molsieve 5A column (pressure: 200 kPa, split flow: 20 ml min^-1^, oven temperature: 80°C) and a RT-Q-bond column (pressure: 150 kPa, split flow: 10 ml min^-1^) with a PDD detector at 110°C. For samples with H_2_ and methane concentrations above 1%, measurements were done on another Compact GC 12 4.0 (Global Analyser Solutions, Breda, Netherlands) with a Molsieve 5A column (operated at 100°C) coupled to a Carboxen 1010 pre-column and a Rt-Q-BOND column (operated at 80°C) with a thermal conductivity detector. Quantification of CH_4_ and H_2_ was done using standards with known concentration.

#### Organic Acids

Liquid samples were taken using a sterile N_2_ flushed syringe and needle and were centrifuged for 10 min at 14000 *g* at 4°C and stored at -20°C until further processing. Samples were again centrifuged for 10 min at 14000 *g* at 4°C and organic acids in the supernatant were analyzed using a Dionex Ultimate 3000RS (Thermo Fisher Scientific, Sunnyvale, CA, United States) equipped with a Phenomenex Rezex Organic Acid H+ column (300 mm × 7.8 mm) (Phenomenex, Torrance, CA, United States). The system was operated at a column temperature of 80°C and a flowrate of 0.5 mL min^-1^. Eluent consisted of 2.5 mM sulfuric acid. Detection was done using a UV detector at 210 nm.

#### Sulfide

For sulfide analysis, liquid samples were directly fixed 1:1 in zinc acetate (5% w/v). Samples were vortexed thoroughly and further diluted when necessary in MQ water. Sulfide measurements were done as described previously (Timmers et al., 2015).

### Thermodynamic Analysis

The Gibbs free energy changes of the different redox reactions that sustain microbial growth in the enrichment culture incubations were calculated. Gibbs free energy changes under standard conditions were calculated according to the tabulated Gibbs free energy of formation values (Thauer et al., 1977). Actual Gibbs free energy changes were calculated from the *in situ* concentrations of the reactants in the different incubations. To account for the elevated sodium bicarbonate/carbonate concentration in the media, activity electrolyte correction factors were estimated according to Buffle, 1988 (estimated *f*-values are 0.5 and 0.04 for monovalent and bivalent ions, respectively). Equilibrium was assumed in the gaseous carbon dioxide, bicarbonate, carbonate system, as well as the gas-liquid partitioning of carbon dioxide, methane, and molecular H_2_. Initially, thermodynamic equilibrium (Δ*G* = 0) was furthermore assumed in the formate to bicarbonate/H_2_ conversion in order to estimate the actual formate concentrations in the system. Henry coefficients that are not corrected for the elevated salt concentration, give unrealistic values for the Gibbs energy change for both H_2_ producing and consuming reactions. To account for the decrease in solubility of H_2_ at 1 M sodium bicarbonate/carbonate, an activity correction for gaseous H_2_ was introduced and roughly estimated to amount to 0.2, according to Engel et al. (1996).

### DNA Isolation and Sequencing of Methanogenic SAO Enrichment Cultures

Methanogenic SAO cultures were used for metagenome sequencing. Cultures were pre-grown on 60 mM acetate. When 30 mM acetate was utilized, cells were centrifuged (1 h, 4,700 *g*) and washed three times in 1x PBS with 1 M NaCl (to remove Na^+^-carbonates). Cells were resuspended in 1x TE buffer and 0.4 mg L^-1^ of polyadenylic acid was added to coat surfaces for prevention of sorption of nucleic acids. Then, lysozyme solution (Masterpure Gram-positive DNA purification kit, Epicentre, Madison, WI, United States) was added and incubated for 30 min at 37°C. Next, proteinase K in Gram-positive lysis solution (Masterpure Gram-positive DNA purification kit) was added and samples were incubated for 15 min at 67°C and vortexed every 5 min. Samples were cooled down to 37°C and placed on ice for 5 min. Then, 1 volume of phenol:chloroform:isoamyl alcohol (24:24:1, v/v/v) was added to the samples and mixed by inversion. Samples were centrifuged at 12000 × *g* for 2 min at room temperature. The aqueous upper layer was transferred to a new tube and nucleic acids were precipitated by adding 1 volume of isopropanol and 0.1 volume of 3 M sodium acetate (pH 5.2). Samples were kept on ice for 20 min and centrifuged at 12,000 × *g* for 20 min at 4°C. The supernatant was removed and the pellet was rinsed with 70% cold ethanol. Tubes were inverted and air dried. Afterwards, 1x TE buffer with RNAse (Masterpure Gram-positive DNA purification kit) was added to the samples and incubated at 37°C for 30 min and nucleic acids were again precipitated by adding 1 volume of isopropanol and 0.1 volume of 3 M sodium acetate (pH 5.2). Samples were kept on ice for 20 min and centrifuged at 12,000 *g* for 20 min at 4°C. The supernatant was removed and the pellet was rinsed with 70% cold ethanol. Tubes were inverted and air dried. The pellet was dissolved in 20 μl DNAse free water. The DNA quality and quantity were checked using the Nanodrop 2000 and the Qubit fluorometer (Thermo Fisher Scientific, Waltham, MA, United States). Samples were sent for sequencing using the Illumina HiSeq 4000 platform (GATC Biotech, Konstanz, Germany).

### Metagenome Analysis of Methanogenic SAO Enrichment Cultures

Raw sequence reads were quality and length (minimum 21 b) trimmed using the software program Sickle (version 1.33) (Joshi and Fass, 2011). Trimmed reads were assembled into contigs >1 kb with MEGAHIT (Li et al., 2015), using the default sensitive mode for generic metagenomes. ORF calling was done with Prodigal (Hyatt et al., 2010), tRNA prediction with tRNAscan-SE (Lowe and Eddy, 1997), rRNA prediction using rna_hmm3. Protein homology searches were performed against the COG (Tatusov et al., 2000) and TIGRFAMs (Haft et al., 2001) databases with predictions of hmmer3 (Eddy, 2011). Best-hits of the CDS and rRNA genes were obtained against the NCBI non-redundant BLAST database (USEARCH, *e*-value = 0.00001) and SILVA (MEGABLAST, *e*-value = 0.00001), respectively.

Archaeal and bacterial contigs were separated based on the taxonomic annotation of the encoded CDS. Contigs that contained genes from both kingdoms were manually inspected and disregarded when they contained less than three genes, were considerably mixed in phylogeny or were from phage/viral origin. The contigs were further binned with MaxBin (version 2.2.1: (Wu et al., 2014)) using 40 and 107 universal marker genes for *Archaea* and *Bacteria*, respectively. MaxBin can infer contig abundance after mapping reads to the contig with Bowtie2 (version 2.2.3) (Langmead and Salzberg, 2012).

The resulting bins were evaluated using CheckM (Parks et al., 2015), abundance information and inspected manually based on the annotated CDS. Three *Euryarchaeota*-, three *Firmicutes*- and two *Deltaproteobacteria*-related bins of reasonable quality (completeness 83–98%, contamination 2–23%) were obtained with estimated coverage >10×. Several very low-abundance bins of mixed phylogeny were also obtained, but excluded from further analysis.

The eight bins of interest were further optimized in iterative steps until CheckM results stagnated after the third reassembly: we checked manually the bins for contamination and spurious contigs, re-assembled with MEGAHIT (–kmin 21, –k-max 121, –k-step 10, –min-contig-len 1000) the subsets of mapped reads to the bins from the previous round and their respective mates, we annotated the contigs and binned archaeal and bacterial contigs separately with MaxBin. After the three iterative binning rounds, 16S rRNA genes were blasted against NCBI-nr (blastn, *e*-value ≤ 0.00001) and manually placed in the correct bins guided by taxonomic gene contig annotations. VizBin [default settings; (Laczny et al., 2015)] was used for further inspection and refinement of the selected bins based on PCA plots (Supplementary Figure S1). Finally, taxonomic assignments of the three *Firmicutes*-related MAGs were double-checked with maximum-likelihood phylogenetic trees constructed with 16S ribosomal proteins as described previously (Hug et al., 2016; Vavourakis et al., 2018), including all 36 available closely related NCBI reference (draft) genomes (Supplementary Figures S2, S3).

Characteristics and taxonomic assignments of the final eight metagenome-assembled genomes (MAGs) are summarized in Supplementary Tables S1, S2. The dominant populations in the enrichment culture belonged to the SAOB (MSAO_Bac1 with 100% 16S rRNA gene identity to ‘*Ca.* S. acetioxidans’ clone AAS1) and its syntrophic methanogenic partner (MSAO_Arc1 with 100% 16S rRNA gene identity to *Methanocalculus* sp. strain AMF5).

The draft genome of ‘*Ca.* S. acetioxidans,’ MSAO_Bac1 (Table 1), was re-annotated against KEGG [Automatic Annotation Server (KAAS)] (Moriya et al., 2007) and RAST (Rapid Annotation using Subsystems Technology) (Aziz et al., 2008). Manual blast runs of translated gene sequences were done using the NCBI’s BLASTP service. Gene domain analysis was done using NCBI’s CDD (Marchler-Bauer et al., 2009) and EMBL’s Interpro (Jones et al., 2014) services. The location of proteins (extra-, intracellular or transmembrane) were checked using PROTTER (Omasits et al., 2014), the TMHMM server v. 2.0 (Sonnhammer et al., 1998) and the SignalP 4.1 Server (Petersen et al., 2011). Genome completeness and redundancy were estimated also based on universal copy genes (Rinke et al., 2013) using the Anvi’o platform (v4; Eren et al., 2015).

**Table 1:** Details on the draft genome of ‘*Ca.* Syntrophonatronum acetioxidans MSAO_Bac1’ (accession number pending) reconstructed from the methanogenic SAO enrichment culture.

Recovered genome size	1.97 Mb
Estimated sequence coverage	665×
G+C content	44.3%
Anvi’o/CheckM completeness	96/90%
Number of tRNA sequences	39
Number of 5S rRNA genes	1
Number of 16S rRNA genes	1
Number of 23S rRNA genes	1
Number of contigs	300
Number of coding sequences	1,876
Coding density	91.7%
Anvi’o redundancy/CheckM contamination	8/12%
CheckM strain heterogeneity	41%
Besthit NCBI-nr 16S rRNA gene	*“Ca.* Syntrophonatronum acetioxidans clone AAS1,” partial sequence
Identity	100%
*e*-value	0
Besthit NCBI-nr gene contigs	*Dethiobacter alkaliphilus*
Of total hits	25%


For comparative SAO analysis, all genomes of SAOBs so far described and some acetogens were compared to MSAO Bac1, including *Acetobacterium woodii* DSM 1030 (NC_016894) (Poehlein et al., 2012), *Clostridium ultunense* Esp (HG764817) (Manzoor et al., 2013b), *Tepidanaerobacter acetatoxydans* Re1 (NC_019954) (Manzoor et al., 2013a), *Syntrophaceticus schinkii* strain Sp3 (NZ_CDRZ01000250) (Manzoor et al., 2015, 2016), *Pseudothermotoga lettingae* (NC_009828) (Zhaxybayeva et al., 2009), and *Thermacetogenium phaeum* DSM 12270 strain PB (bioproject PRJNA168373) (Oehler et al., 2012).

To estimate the relative abundance of MSAO_Bac1 in native soda lake sediments, a recruitment experiment was performed using 10 million reads subsamples from previously described metagenomes obtained from soda lake sediments in Kulunda Steppe in the summer of 2010 and 2011 (Vavourakis et al., 2018). Maximum likelihood phylogeny was calculated based on 16 ribosomal proteins predicted in selected *Syntrophomonadaceae* reference genomes available at the time of analysis (NCBI) and in MSAO_Bac1 as described previously (Vavourakis et al., 2018). Species delineation was determined with Average Nucleotide Identities [ANI, (Goris et al., 2007)]. We further used ANI and CheckM results to select only one representative MAG for each species and re-calculated the phylogeny of the 16 ribosomal proteins. The final phylogenetic tree, results of the recruitment experiment and genome annotation [Ghost Koala; (Kanehisa et al., 2016)] summaries were visualized using iTOL v4 (Letunic and Bork, 2007).

### Lipid Analysis of Methanogenic SAO Enrichment Cultures

Lipids were extracted with a Bligh Dyer method and the extract was acid hydrolyzed. The resulting core lipids were methylated and silylated and analyzed by gas chromatography and gas chromatography-mass spectrometry. The Bligh Dyer extract was also analyzed directly for intact polar lipids using liquid chromatography-mass spectrometry. All methods have been previously described in detail (Sinninghe Damsté et al., 2011).

## Results and Discussion

### Activity of Methanogenic and Sulfate-Reducing SAO Enrichment Cultures

**FIGURE 1:**
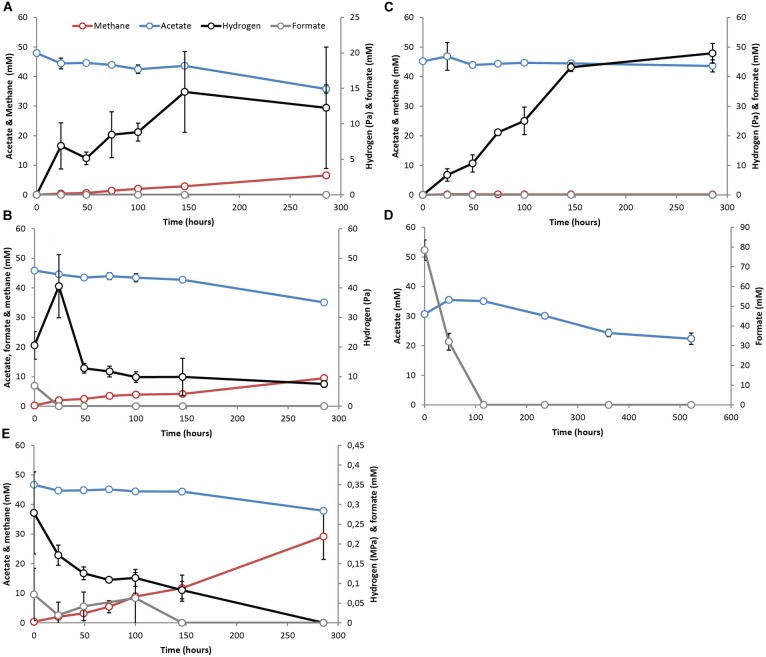
Activity test results with pre-grown syntrophic acetate oxidizing cultures with a methanogenic partner (M-SAO) showing acetate (blue line), H_2_ (black line), formate (gray line) and methane (red line) evolution in cultures supplemented with **(A)** acetate, **(B)** acetate and formate – 7 mM, **(C)** acetate and BES, **(D)** acetate and formate – 80 mM, **(E)** acetate and 100% H_2_. Standard deviations represent biological triplicate incubations.

**FIGURE 2:**
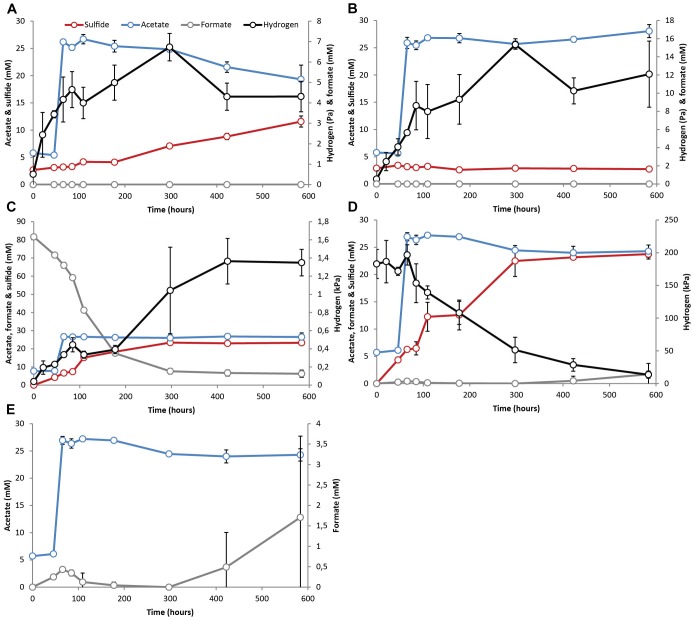
Activity test results with pre-grown syntrophic acetate oxidizing cultures with a sulfate-reducing partner (S-SAO) showing acetate (blue line), H_2_ (black line), formate (gray line) and sulfide (red line) evolution in cultures supplemented with **(A)** acetate, **(B)** acetate and molybdate, **(C)** acetate and formate – 80 mM, **(D)** acetate and 100% H_2_ showing acetate, H_2_ and sulfide and **(E)** acetate and 100% H_2_ showing only acetate and formate evolution. Standard deviations represent biological triplicate incubations.

The methanogenic SAO (M-SAO) and sulfate reducing SAO (S-SAO) enrichment cultures degraded acetate with coinciding H_2_ production and subsequent methane or sulfide production, respectively. Hydrogen accumulated up to 10.7 (±0.6) and 12.3 (±8.6) Pa in static and shaken M-SAO cultures (Figure 1A and Supplementary Figure S4A, respectively) and to 4.3 (±0.7) and 5.1 (±1.0) Pa in static and shaken S-SAO cultures (Figure 2A and Supplementary Figure S4B, respectively). The lower H_2_ concentration in the S-SAO cultures suggests that SRB are capable of achieving a lower H_2_ partial pressure as compared to methanogens. SAO was apparently energetically feasible at H_2_ concentrations higher than the values calculated for neutrophilic SAOBs (Dolfing, 2014). The actual Gibbs free energy change of acetate oxidation is energetically unfavorable if one considers the H_2_ solubility in pure water (Table 2, ΔG^1^). However, when a H_2_ solubility of 20% of the solubility in pure water is assumed, due to the *in situ* sodium bicarbonate/carbonate concentrations, acetate oxidation becomes favorable (Table 2, ΔG^1∗^). Using this assumption, the Gibbs free energy changes of conversion for both partner organisms are similarly negative at the rather stable H_2_ partial pressures in these cultures, indicating that the syntrophic partners equally share the energy gained from the total reaction (Table 2). Formate was never detected (Figures 1A, 2A and Supplementary Figures S4A,B). Assuming that formate conversion to H_2_/CO_2_ is at thermodynamic equilibrium, the calculated formate concentrations from the maximum H_2_ concentrations in these incubations are in the range of 10 μM, which was always below the detection limit of 50 μM. Therefore, a role of formate cannot be excluded.

**Table 2:** Thermodynamic calculations according to the measured parameters in the incubations that performed syntrophic acetate oxidation coupled to methanogenesis (M-SAO) or sulfate reduction (S-SAO) with and without inhibitors bromoethanesulfonate (BES) or Molybdate ($MoO_{4}^{2-}$), respectively.

M-SAO cultures	Reaction	ΔG^1^ (kJ mol^-1^)	ΔG^1∗^ (kJ mol^-1^)
Acetate oxidation	CH_3_COO^-^ + 4 H_2_O → 2 ${HCO}_{3}^{-}$ + 4 H_2_ + H^+^ [H_2_] = 11.5 Pa	+2.2	-13.7
Hydrogenotrophic methanogenesis	4 H_2_ + ${HCO}_{3}^{-}$ + H^+^ → CH_4_ + 3 H_2_O [H_2_] = 11.5 Pa	-31.2	-15.3
Total	CH_3_COO^-^ + H_2_O → ${HCO}_{3}^{-}$ + CH_4_ [H_2_] = 11.5 Pa	-29	-29
Acetate oxidation + BES	CH_3_COO^-^ + 4 H_2_O → 2 ${HCO}_{3}^{-}$ + 4 H_2_ + H^+^ [H_2_] = 47.9 Pa	+16.3	+0.4
**S-SAO cultures**			
Acetate oxidation	CH_3_COO^-^ + 4 H_2_O → 2 ${HCO}_{3}^{-}$ + 4 H_2_ + H^+^ [H_2_] = 4.7 Pa	-6.2	-22.3
Hydrogenotrophic sulfate-reduction	4 H_2_ + ${SO}_{4}^{2-}$ + H^+^ → HS^-^ + 4 H_2_O [H_2_] = 4.7 Pa	-33.3	-17.3
Total	CH_3_COO^-^ + ${SO}_{4}^{2-}$ → 2 ${HCO}_{3}^{-}$ + HS^-^ [H_2_] = 4.7 Pa	-39.5	-39.6
Acetate oxidation + $MoO_{4}^{2-}$	CH_3_COO^-^ + 4 H_2_O → 2 ${HCO}_{3}^{-}$ + 4 H_2_ + H^+^ [H_2_] = 13.7 Pa	+4.4	-11.6


*Gibbs free energy calculations were conducted assuming a Henry coefficient of 1.7 10^-*4*^ mol/L/Atm as defined for pure water (ΔG^*1*^) and a five time lower value to account for the decreased solubility in a 1 M sodium bicarbonate/carbonate medium (ΔG^*1*∗^).*

*ΔG^*1*^ is calculated using media conditions, assuming bicarbonate/carbonate/and gaseous carbon dioxide in equilibrium, and formate in equilibrium with bicarbonate and gaseous H_*2*_. The resulting average concentrations are with activity correction according to Buffle (1988) (with small variations between experiments): acetate 60 mM, pH 9.5, P_*CO*_*2*__ 0.01 bar, ${HCO}_{3}^{-}$ 0.65 M, ${CO}_{3}^{2-}$ 0.17 M, formate 0.1 mM. Average H_*2*_ concentrations were estimated from the stable concentrations in the incubations. ΔG^*1*∗^ is calculated from ΔG^*1*^ with an activity correction of 0.2 for gaseous H_2_ solubility, according to Engel et al. (1996).*

There was no significant difference in acetate consumption and H_2_, methane or sulfide formation when cultures were shaken or statically incubated (Figures 1A, 2A, and Supplementary Figures S4A,B). This indicates that IEC transfer is not influenced by complete mixing. Microscopic investigations showed no physical association of partner organisms (Sorokin et al., 2014a, 2016). Aggregation or close association is not essential when H_2_ and/or formate act as IEC (Stams and Plugge, 2009).

When the methanogenic inhibitor 2-bromoethanesulfonate (BES) was supplied to M-SAO cultures, only 1.6 mM (±1.7) of acetate was consumed and H_2_ accumulated up to 47.9 (±3.3) Pa (Figure 1B). In the S-SAO cultures, the sulfate-reducing inhibitor molybdate ($MoO_{4}^{2-}$) inhibited acetate oxidation and sulfide production and H_2_ accumulated until between 12.1 (±3.6) and 15.3 (±0.4) Pa (Figure 2B). Acetate oxidation at these H_2_ concentrations was indeed energetically less favorable for S-SAO and even unfavorable for M-SAO cultures (Table 2). The calculated concentration of H_2_-derived formate from the maximum measured H_2_ concentration at thermodynamic equilibrium is 35 μM when BES was added and 26 μM when molybdate was added, which are below the detection limit of formate.

When formate was supplied together with acetate, formate was consumed in both M-SAO and S-SAO cultures with concomitant increase in H_2_ and subsequent methane or sulfide production, respectively (Figures 1C, 2C). This indicates that formate was first converted to H_2_ before being consumed by the syntrophic partner. In the M-SAO cultures when only 7 mM formate was supplied, formate was consumed rapidly which resulted in a H_2_ peak of 40.6 (±10.7) Pa and stoichiometric methane formation of 1.8 (±0.2) mmol per liter media (Figure 1C). When 80 mM formate was supplied to both cultures, formate was consumed and no acetate consumption occurred if formate was present (Figures 1D, 2C). Only when all formate was consumed after 115.5 h, acetate oxidation started and 12.7 mM (±1.4) acetate was consumed after 522 h (Figure 1D, methane and H_2_ data not shown). In S-SAO cultures, formate consumption resulted in an increase in H_2_ to a total of 1.3 (±0.1) kPa and sulfide to a total of 23.3 mM (±0.9) (Figure 2C). After 300 h, residual formate was slowly consumed and no acetate consumption was noted during the whole incubation time, probably because not all formate was consumed, as opposed to the M-SAO cultures.

When 100% H_2_ was added together with acetate, H_2_ was consumed to produce methane or sulfide in M-SAO or S-SAO cultures, respectively. Formate was produced in both cultures and its presence inhibited acetate consumption (Figures 1E, 2D,E). The M-SAO cultures only started to consume acetate after 146 h of incubation in parallel with H_2_ consumption. This inhibition of acetate consumption corresponded to the presence of formate during the first 146 h of incubation. When formate levels decreased, acetate was consumed again (Figure 1E). In S-SAO cultures, H_2_ consumption corresponded to sulfide production (Figure 2D). Acetate consumption was also inhibited when formate was produced after 297 h (Figure 2E). Mass transfer limitation of externally supplied gaseous H_2_ in these static incubations probably explains the different inhibitory effects of H_2_ and formate.

Hydrogen-formate interconversion occurred in both M-SAO and S-SAO cultures. Pure cultures of the methanogenic partner *M. natronophilus* and of the sulfate reducing partner *D. magnus* growing on H_2_ also produced formate and vice versa (Supplementary Figure S5). This indicates that *M. natronophilus* and *D. magnus* might be responsible for H_2_-formate interconversion in the SAO cultures. Interestingly, SAO rates were four times higher with *M. natronophilus* as partner than with *D. magnus* as partner (acetate consumption of 0.04 mM h^-1^ vs. 0.01 mM h^-1^, respectively) although the Gibbs free energy change for S-SAO was higher (Table 2). Pure cultures of *M. natronophilus* showed much faster methane production and growth on H_2_ than on formate, whereas *D. magnus* showed the opposite for sulfide production and growth (Supplementary Figure S6). The assumption that H_2_ is probably the main IEC in SAO would explain the faster SAO rates with a *M. natronophilus* as partner. Overall, the data provides no answer to the question if formate and/or H_2_ is the actual IEC, but do suggest that both are largely equivalent and interchangeable.

### Genetic Potential of ‘*Ca.* S. Acetioxidans’

The metagenome of the M-SAO enrichment culture consisted of eight metagenome-assembled genomes (MAGs) (Supplementary Figure S1 and Supplementary Tables S1, S2). The dominant populations in the enrichment culture belonged to the SAOB (MSAO_Bac1), with 100% 16S rRNA gene identity to ‘Ca. S. acetioxidans’ clone AAS1, and its syntrophic methanogenic partner (MSAO_Arc1), with 100% 16S rRNA gene identity to *Methanocalculus natronophilus* strain (AMF5). Details on the draft genome of ‘Ca. S. acetioxidans’ are given in Table 1. According to the genome completeness and redundancy estimates obtained through Anvi’o, and the presence of rRNA and tRNA genes, our MAG can be viewed as a high-quality draft (Bowers et al., 2017).

#### Hydrogenases and Formate Dehydrogenases

The partial genome of ‘*Ca.* S. acetioxidans’ encodes two [NiFe] hydrogenases. One is a homolog to the *Escherichia coli* Hya hydrogenase which consists of a large and small cytochrome *c*3-containing subunit and a cytochrome *b* subunit. Only the small cytochrome *c*3 subunit has a twin-arginine translocation (TAT) signal and is anchored in the membrane. The cytochrome *b* was predicted to be transmembrane (k121-2698, [NiFe] hydrogenase 1). The TAT system can transport the complete hydrogenase dimer across the membrane when the TAT signal peptide is located only on one of the subunits via a so-called hitchhiker mechanism (Rodrigue et al., 1999). The active site on the large subunit is therefore probably extracellularly oriented and involved in H_2_ oxidation or production and electron transfer from or to menaquinone via cytochrome *b*. The second, [NiFe] hydrogenase 2, (k121-3777) consists only of a large and small cytochrome *c*3-containing subunit without signal peptides to translocate it over the cell membrane. The small subunit is anchored in the membrane, probably intracellularly. Such ‘group 5’ [NiFe] hydrogenases are involved in H_2_ oxidation at very low H_2_ concentrations (Peters et al., 2015). Most SAOB described so far encode for [FeFe] hydrogenases that are involved in H_2_ production (Manzoor et al., 2018). A cytoplasmic [NiFe] hydrogenases was also found to be expressed by *S. schinkii* during SAO, and was thought to be involved in intracellular H_2_ sensing for subsequent energy-transducing reactions since the gene was located next to a response regulator receiver gene (Manzoor et al., 2016). In ‘*Ca.* S. acetioxidans,’ the [NiFe] hydrogenases 2 genes surround a methylenetetraydrofolate reductase gene (*MetF*) and are probably directly involved in acetate metabolism (see section “Energy Conservation”), either in regulation of gene expression of other hydrogenases in response to SAO, or to produce H_2_ during SAO.

The partial genome of ‘*Ca.* S. acetioxidans’ also contains several formate dehydrogenases (FDHs). Three of them contain 4Fe-4S ferredoxin-containing beta subunits (k121-3286-cds8, k121-4883-cds7 and k121-6301-cds1). Most FDHs seem to have the active site intracellularly and formate and H_2_ can therefore be interconverted in the cytoplasm. One FDH alpha subunit has a TAT signal, indicating that it is exported and thus might be involved in extracellular formate conversion (k121-1471-cds2). The genome does not encode formate transporters and it therefore seems most plausible that the SAOB produces H_2_ as an end product during SAO. Yet, it could potentially convert H_2_ to formate outside of the cell, using extracellular [NiFe] hydrogenases and formate dehydrogenases.

#### Acetate Activation and Uptake

**FIGURE 3:**
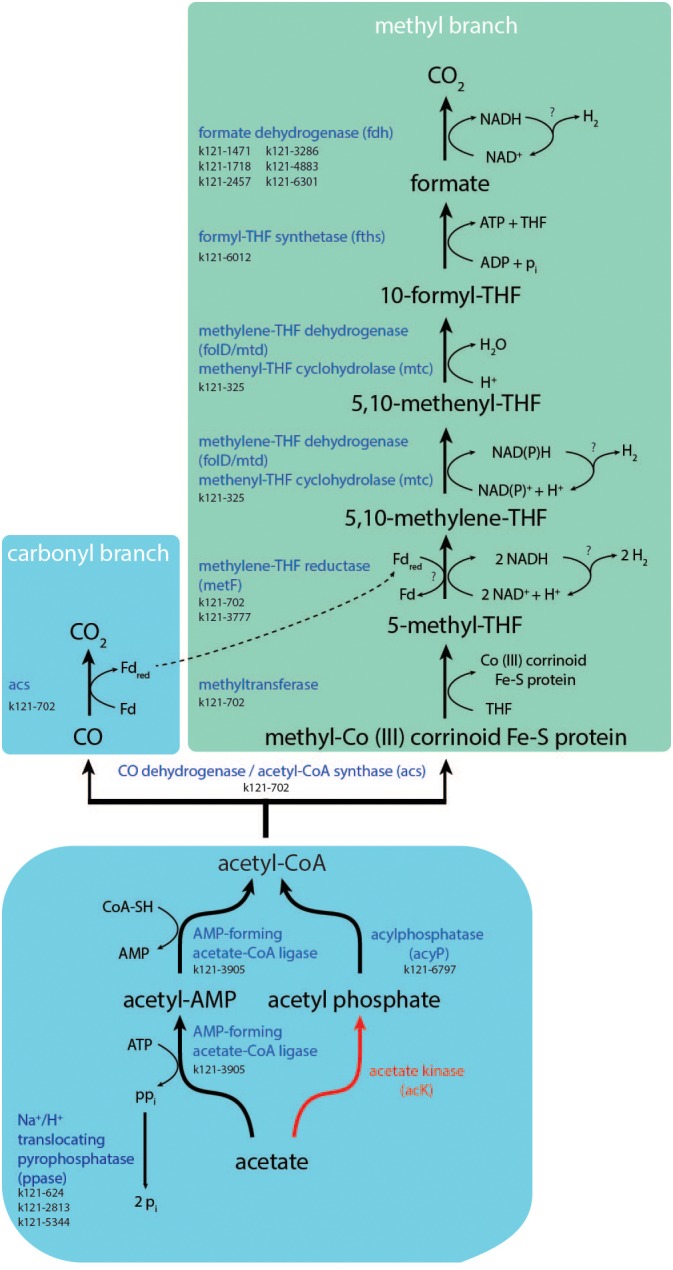
Summary of the Wood–Ljungdahl pathway and acetate activation. Red lines and red names indicate absence of these conversions or genes in the partial genome of ‘*Ca.* Syntrophonatronum acetioxidans.’

**FIGURE 4:**
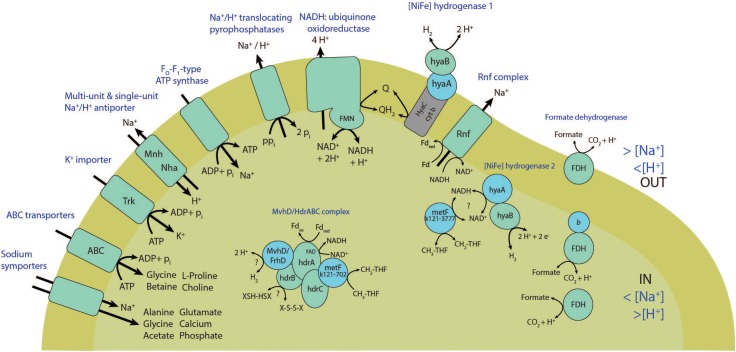
Scheme showing transmembrane transporters, energy conserving enzymes and electron transport mechanisms found in the genome of ‘*Ca.* Syntrophonatronum acetioxidans.’

‘*Ca.* S. acetioxidans’ does not contain the conventional acetate kinase (*ACK*) gene for activating acetate to acetyl phosphate (acetyl-P) that was found in genomes of the previously characterized SAOBs *Pseudothermotoga lettingae* strain TMO (Zhaxybayeva et al., 2009), *Tepidanaerobacter acetatoxydans* strain Re1 (Müller et al., 2015), *Syntrophaceticus schinkii* strain Sp3 (Manzoor et al., 2016), *Thermacetogenium phaeum* strain DSM 12270 (Oehler et al., 2012), and *Clostridium ultunense* strain Esp (Manzoor et al., 2013b). It also does not contain the gene for phosphotransacetylase (*PTA*), that further converts acetyl-P to acetyl-CoA. The enzymes PTA and ACK are mainly operational at high acetate concentrations. ‘*Ca.* S. acetioxidans’ does have the gene that encodes for acylphosphatase (*acyP*) that could also produce acetyl-CoA from acetyl-P (Figure 3). For acetate activation, it does have genes encoding AMP-forming acetate-CoA ligase (k121-3905) that catalyzes the conversion of acetate to acetyl-CoA via acetyl-AMP in *Bacteria* and *Archaea* for anabolic acetate assimilation. It is reversible *in vitro*, but the reaction is irreversible *in vivo* with presence of intracellular pyrophosphatases (Wolfe, 2005). It, therefore, serves as the main route for acetate assimilation at low acetate concentrations since the enzyme was considered to have a high affinity for acetate with a low activity (Starai et al., 2003; Berger et al., 2012). In its natural environment, an acetate oxidizer must cope with low acetate concentrations, which corresponds to the high affinity acetate activation via AMP-forming acetate-CoA ligase. It also has genes for an AMP-forming phenylacetate-CoA ligase (k121-6797/k121-3180) that can activate phenylacetate. This enzyme also acts, albeit less specifically, on acetate, propionate and butyrate (Martinez-Blanco et al., 1990) and could therefore also be responsible for acetate activation during SAO. Since very little energy can be conserved from SAO, it is surprising that ‘*Ca.* S. acetioxidans’ only has a gene for the AMP-forming acetyl-CoA synthetase because this enzyme requires two ATP molecules per molecule of acetate whereas the ACK/PTA system requires only one ATP (Berger et al., 2012). However, it is possible that the generated pyrophosphate (PP_i_) is not completely hydrolyzed by pyrophosphatases and a part is used for transfer of phosphate groups to other intermediates, thereby conserving some energy from the reaction (Berger et al., 2012). Multiple genes were indeed encoding H^+^/Na^+^ translocating pyrophosphatases (Figures 3, 4 and Supplementary Data S1) that hydrolyse PP_i_ for proton or sodium translocation and thereby possibly creating a proton or sodium motive force, respectively (Baykov et al., 2013). This mechanism was indeed postulated to be an adaptation to low energy supply (Luoto et al., 2013) and has been found in all other SAOBs described so far (Manzoor et al., 2018).

The gene for an acetate-transporting permease (*ActP*) is often found to cluster with acetyl-CoA synthetase (AMP-forming) gene (Gimenez et al., 2003). However, *ActP* was not present in the genome, nor was it present in any other SAOB genome in our comparison. We found a putative Na^+^/solute symporter (k121-3905) encoded next to the gene for acetyl-CoA synthetase (AMP-forming). The genome of another SAOB, *S. schinkii* Sp3, also contained a gene related to Na^+^/solute symporters and this gene was previously identified to encode for an acetate transporter in *E. coli* (Manzoor et al., 2016). Genome analysis of *T. acetatoxydans* also showed presence of Na^+^/solute symporters (Müller et al., 2015).

#### The SAO Pathway

Most SAOBs described so far cluster with the physiological group of homoacetogens, which possess the Wood–Ljungdahl (WL) pathway and experimental evidence indicated that indeed this pathway is used in reverse for SAO by at least *T. phaeum* and *C. ultunense* (Schnürer et al., 1997; Hattori et al., 2005; Oehler et al., 2012). The partial genome of ‘*Ca.* S. acetioxidans’ contains all genes that encode for the (reverse) WL pathway to oxidize acetyl-CoA (Figure 3) and it likely uses this route for acetyl-CoA oxidation. An alternative route using the oxidative TCA cycle was suggested for *T. acetatoxydans* (Müller et al., 2013). ‘*Ca.* S. acetioxidans’ has an incomplete TCA cycle as it lacks the genes for conversion of malate to oxaloacetate and citrate to isocitrate (Supplementary Figure S7 and Supplementary Discussion). Therefore, it seems unlikely that it oxidizes acetate through this pathway.

#### Energy Conservation During SAO

The reversal of the WL pathway generates several problems for energy conservation. Firstly, it creates only one ATP during formyl-tetrahydrofolate synthase activity, but acetate activation costs two ATP. Energy must therefore come from the generation of a sodium or proton motive force during SAO. The partial genome of ‘*Ca.* S. acetioxidans’ encodes for an F_1_F_O_ -type ATP synthase. Multiple sequence alignment shows that the c-subunit of the F_1_F_O_-ATP synthase of ‘*Ca.* S. acetioxidans’ (k121-2696) has conserved amino acids involved in Na^+^-binding that are present in Na^+^-dependent F_1_F_O_ -ATP synthases but not in H^+^-dependent F_1_F_O_ -ATP synthases (Mulkidjanian et al., 2008; Oehler et al., 2012; Schulz et al., 2013) (Supplementary Figure S8). Na^+^-dependent ATP synthases provide an advantage at haloalkaline conditions where extracellular Na^+^ concentrations are high and thus contribute to the sodium motive force (Figure 4). To establish a sodium motive force, the Na^+^ levels in the cell need to be kept lower than externally. As mentioned above, the genome contains H^+^/Na^+^ translocating pyrophosphatases that produce a sodium motive force during acetate activation. Secondly, the genome contains genes encoding for Na^+^ efflux proteins and single- and multisubunit Na^+^/H^+^ antiporters (see section “Adaptations to Haloalkaliphilic Conditions”).

The genome encoded for two transmembrane complexes that combine production of a sodium motive force to NADH recycling and subsequent H_2_ production. Three subunits of the Rnf complex (Na^+^-translocating ferredoxin: NAD^+^ oxidoreductase) were encoded in the genome; subunits C, D and two genes for subunit G (Supplementary Data S1). Both genes for subunit G contained a signal peptide for export and *RnfD* was transmembrane. Subunit RnfC is directly involved in NADH recycling that drives RnfD to pump Na^+^ or H^+^ ions. These genes are homologs to the Na^+^-pumping NADH:quinone oxidoreductase (Na^+^-NQR), but multiple sequence alignment showed that they are more related to Rnf complex genes (data not shown). *T. phaeum* contains only one gene with weak similarity to the subunit *RnfC* (Oehler et al., 2012) and in *S. schinkii* transcription levels of the Rnf complex were very low when grown syntrophically on acetate (Manzoor et al., 2016). This could imply that the Rnf complex has no significant role in energy conservation during SAO, but this needs to be investigated for ‘*Ca.* S. acetioxidans’. Lastly, two contigs of the genome encoded for NADH:ubiquinone oxidoreductase subunits NuoEFGI (Supplementary Data S1). These subunits form the soluble fragment and catalyze the oxidation of NADH (Braun et al., 1998). Since we did not find the hydrophobic membrane fragment in the draft genome (*NuoAHJKLMN* subunits) (Leif et al., 1995), it is not clear if the ‘*Ca.* S. acetioxidans’ genomes encodes for the whole complex or if genes were missed by incompleteness of the draft genome (Table 1). Still, since it was proposed that acetogens can be bioenergetically classified into Rnf and Ech-containing groups with either Na^+^ and H^+^-dependence (Hess et al., 2014), we propose to classify ‘*Ca.* S. acetioxidans’ as a Rnf-containing Na^+^-dependent acetogen.

The second challenge for energy conservation during SAO is the production of NADH (E° = -320 mV) from methyl-THF oxidation (*E*° = -200 mV). This conversion is endergonic under standard conditions. Therefore, a bifurcation mechanism that couples this to a favorable conversion is expected. Interestingly, ‘*Ca.* S. acetioxidans’ contained two copies of the gene coding for methylenetetrahydrofolate (methylene-THF) reductase (metF). One was located in the same contig with the genes for CODH/acetyl-CoA synthase and methyltetrahydrofolate:corrinoid methyltransferase (*acsE/metH*) (k121-702). Besides these genes, this *acs* operon also contains genes for a MvhD/HdrABC-like complex. Strangely, the genes coding for the HdrA subunit contains a TAT signal peptide for export and is next to the TAT protein translocase system (*TatABC*). The subunits HdrB and HdrC were encoded on a different contig and are predicted to be cytoplasmic (k121-4746). In the acetogen *Moorella thermoacetica*, *metF* and *HdrABC/MvhD* form a transcript (Mock et al., 2014). There, the genes encoding homologs of the soluble HdrABC complex, similar to the one of *Methanothermobacter marburgensis*, are downstream of methylene-THF reductase subunits *MetV* and *MetF* genes with a ferredoxin-coding gene in between (Schuchmann and Müller, 2014). Partial purification of this enzyme complex of *M. thermoacetica* showed that it is a heterohexamer of MetFV, HdrABC, and MvhD that uses NADH as electron donor. The complex, however, does not catalyze NADH dependent methylene-THF reduction and does not use ferredoxin as an electron acceptor. It still needs to be investigated if this HdrABC performs electron bifurcation with a second electron acceptor (Mock et al., 2014; Schuchmann and Müller, 2014). In the SAOB *T. phaeum*, the *acs* operon also contained *Hdr*-like genes (Oehler et al., 2012) and a Hdr/NAD-binding oxidoreductase complex was expressed during SAO in *S. schinkii* (Manzoor et al., 2016). In *T. phaeum* it was assumed that a bifurcating hydrogenase was coupled (directly or indirectly via menaquinone) to oxidation of methyl-THF (Oehler et al., 2012; Manzoor et al., 2016). In *Syntrophobacter fumaroxidans*, the Hdr/MvhD complex of that bacterium was detected under sulfate-reducing conditions, whereas only the subunits containing FAD/NAD binding domains were detected under syntrophic conditions (Sedano-Nuñez et al., 2018). CO oxidation to CO_2_ (-520 mV) by the CODH/ACS is coupled to ferredoxin reduction (-450 mV) and is exergonic. Production of NADH (-320 mV) during methyl-THF oxidation (-200 mV) is endergonic. Therefore, we propose a flavin-based electron bifurcating mechanism where the exergonic oxidation of ferredoxin drives the endergonic reduction of NAD^+^ during methyl-THF oxidation (Figure 4), similar as was found in *M. thermoacetica*, but reversed. The other *metF* copy in the genome of the SAOB (k121-3777) is located next to a [NiFe] hydrogenase ([NiFe] hydrogenase 2) and possibly involved in H_2_ production during SAO (see section “Hydrogenases and Formate Dehydrogenases”).

#### Adaptations to Haloalkaliphilic Conditions

At haloalkaline conditions, microorganisms must cope with two extremes, namely high pH and high osmotic pressure. ‘*Ca.* S. acetioxidans’ is an obligate haloalkaliphile as its optimal growth condition is around pH 9.5–10 and 1 M Na^+^ in the form of carbonate/bicarbonate. To be able to thrive at these conditions, the organism needs some specific adaptations to maintain pH homeostasis and osmotic balance. The two strategies for osmotic adaptation are the “salt-in” and “salt-out” strategies. With the “salt-in” strategy, extreme halophilic prokaryotes accumulate K^+^ to balance the high Na^+^ concentrations outside of the cell. We found two genes encoding for K^+^ uptake proteins that belong to the Trk K^+^ transport system (Figure 4 and Supplementary Data S1). Halophilic microorganisms that accumulate KCl are also characterized by an excess of acidic amino acids in their proteins (Oren, 2013). The isoelectric point profile of the predicted proteome of ‘*Ca.* S. acetioxidans’ does not show a pronounced acidic proteome and thus probably employs mostly a “salt-out” strategy (Supplementary Figure S9).

The “salt-out” strategy involves Na^+^ extrusion and organic osmolytes (compatible solutes) production. As mentioned, the partial genome encodes for multiple Na^+^ extrusion mechanisms, such as Na^+^ efflux systems (NatB, k121-1613-cds22) and single subunit (Nha, k121-1712-cds1) and multisubunit Na^+^/H^+^ antiporters (Mnh, k121-5712) for regulation of pH and electronegativity homeostasis. As opposed to the Nha antiporters, Mnh antiporters have larger and more extensive proton gathering funnels, which presumably makes them more suitable for H^+^ scavenging; essential at maintenance of pH homeostasis in haloalkaliphiles (Horikoshi, 2011). Common compatible solutes are L-proline, glycine betaine and ectoine. The partial genome of ‘*Ca.* S. acetioxidans’ encodes most genes necessary for biosynthesis of L-proline. We did not find the complete pathway for choline biosynthesis or for glycine betaine production from choline (Supplementary Data S1), and no genes for ectoine production were present. Glycine, cysteine, and serine are derivatives of 3-phosphoglycerate, which is an intermediate of the glycolysis and pentose-phosphate pathway. The glycolysis, TCA cycle and the non-oxidative branch of the pentose phosphate pathway are all encoded in the genome (Supplementary Figure S7 and Supplementary Discussion). Serine and glycine can be produced from 3-phosphoglycerate (Supplementary Data S1). Glycine can also be produced from serine using serine hydroxymethyl transferase (k121-2696-cds48) or from glyoxylate using alanine-glyoxylate transaminase (k121-3258-cds5). Glycine betaine (trimethylglycine) is produced from glycine betaine aldehyde using betaine aldehyde dehydrogenase (k121-1838-cds1). The glycine betaine aldehyde is normally produced from choline, but no choline dehydrogenases or choline monooxygenases were found in the genome of ‘*Ca.* S. acetioxidans’. Glycine betaine can, however, also be produced from glycine using glycine methyltransferase (k121-2696-cds48) to produce sarcosine, but no genes for sarcosine conversion to glycine betaine were found. However, the genome encodes a putative protein-S-isoprenylcysteine methyltransferase (k121-1838-cds2) next to the betaine aldehyde dehydrogenase that could act as a multifunctional enzyme to convert sarcosine to *N*,*N*-dimethylglycine and subsequently to glycine betaine by producing *S*-adenosyl L-homocysteine from *S*-adenosyl L-methionine. Furthermore, uptake systems for glycine betaine, choline, and L-proline are encoded in the genome (Figure 4 and Supplementary Data S1). The “salt-out” strategy with osmotic solutes allows fast adaptation to rapid fluctuations in salinity (Oren, 2013), which is probably key for survival of ‘*Ca.* S. acetioxidans’ in soda lake environments that have a high fluctuation of salinity due to dry and wet seasons.

Soda lakes have besides a high pH and high sodium carbonate concentrations also very low concentrations of unbound divalent ions (mainly Ca^2+^ and Mg^2+^). The partial genome of the SAOB contains many ABC transporters and several divalent ion uptake systems for magnesium, cobalt, nickel, calcium, zinc, manganese, tungsten and iron (Supplementary Data S1). It also contains a gene encoding an ammonium transporter (*AmtB*, k121-1147-cds13) for ammonium uptake. At haloalkaline conditions, ammonium occurs mainly as the free ammonia which can diffuse through the membrane, but only at high concentrations.

### Membrane Lipid Composition

The lipid composition of the M-SAO enrichment culture showed the presence of two types of core membrane diether lipids – the archaeal lipid archaeol (37%) and bacterial diether lipids (63%) (Supplementary Figure S10). Archaeol is derived from *M. natronophilus* AMF5 as was confirmed by analysis of a pure culture of *M. natronophilus* AMF5. The bacterial diether lipids (63%) comprise the core lipids of the bacterial fraction of the M-SAO enrichment culture, that was highly enriched in ‘*Ca.* S. acetioxidans.’ These are composed of C_31_-C_37_ unsaturated dialkyl glycerol diethers. Their alkyl chains are predominantly composed of C_14_ and C_16_ *n*-alkyl moieties as demonstrated by hydrogenation and subsequent GC-MS analysis: 1-*n*-tetradecyl-2 -*n*-hexadecyl glycerol diether and 1,2-di-*n*-hexadecyl glycerol diether, identified by comparison to literature data (Pancost et al., 2001), were the most prominent hydrogenation products obtained. Fatty acids were only minor fractions of lipids in comparison to the unsaturated diakyl glycerol diethers, indicating that ‘*Ca.* S. acetioxidans’ is producing a highly unusual lipid membrane that is predominantly composed of diether lipids. Analysis of intact polar lipids revealed that the head groups of these diethers was predominantly phosphocholine. The occurrence of phosphocholine headgroups in phospholipids in prokaryotes is not common, but it was reported that around 10% of all bacterial genomes possess the pathways to produce phosphocholine (Sohlenkamp et al., 2003). Choline is also a precursor for the compatible solute glycine betaine. It was hypothesized that choline could be released from phosphocholine by phospholipase under hyperosmotic conditions (Sohlenkamp et al., 2003). For the biosynthesis of intact polar lipids, we indeed only found diacylglycerol kinase (k121-2772-cds2) and phosphatidate cytidylyltransferase (k121-3334-cds5) for conversion of 1,2 diacyl-*sn*-glycerol to CDP-diacyl glycerol, but no genes for its conversion to phosphocholine (Boumann et al., 2006). We did find a lysophospholipase L1 in the draft genome (k121-3840-cds54). This enzyme could catalyze the conversion of 2-lysophosphatidylcholine to glycerophosphocholine which can subsequently be converted to glycerophosphate and choline by a glycerophospho-diesterase (k121-1838-cds3, k121-2762-cds8 or k121-4277-cds1). However, the pathways of phosphocholine synthesis and degradation in prokaryotes need further investigation (Sohlenkamp et al., 2003), as well as the biosynthesis of bacterial dialkyl glycerol diethers (Grossi et al., 2015). Alkyl glycerol ether lipids are typically present in archaeal membranes and ensure lower permeability of ions and higher stability than bacterial membrane polar lipids containing esterified fatty acids (Koga, 2012; Grossi et al., 2015). Bacterial alkyl glycerol ether lipids were found in (hyper)thermophilic bacteria and acidophilic bacteria (Koga, 2012; Siliakus et al., 2017), but also in mesophilic *Planctomycetes* (Sinninghe Damsté et al., 2005) and sulfate-reducing bacteria (Grossi et al., 2015). The function of this uncommon lipids in bacteria is not known, but they probably give the cell membrane a higher degree of stability and impermeability, as was shown for archaeal dialkylglycerol ethers (Grossi et al., 2015). The dominance of ether lipids with phosphocholine headgroups in the membrane of ‘*Ca.* S. acetioxidans’ may reflect an adaptation to the high pH and high salt concentrations, but knowledge on the membrane lipid compositions of close relatives that grow under less extreme condition is required to test this hypothesis.

### The Importance of SAO in Haloalkaline Environments

**FIGURE 5:**
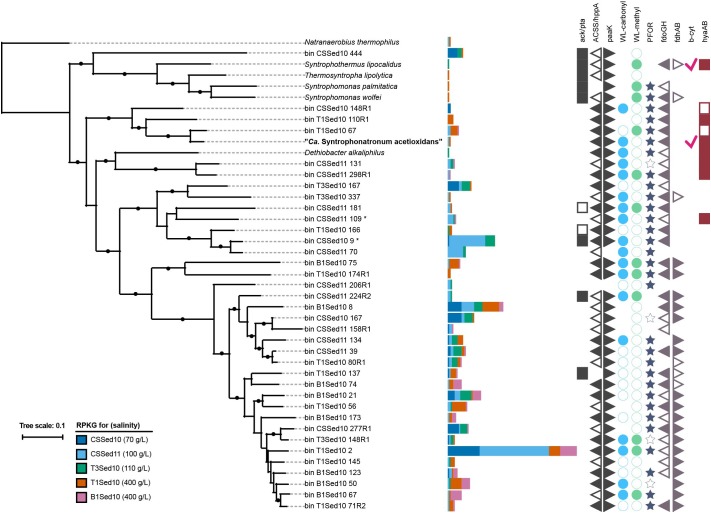
Maximum likelihood tree based on the phylogeny of 16 ribosomal proteins found in selected reference genomes from the family *Syntrophomonadaceae* and the draft genome of “*Ca.* S. acetioxidans” (MSAO_Bac 1). The tree was rooted to the proteins found in *Natranaerobius thermophilus*. The estimated relative abundance of each organism in five different hypersaline soda lake sediment samples is expressed as reads per Kb of genome sequence per Gb of mapped reads (RPGK). Black dots at the nodes show the ≥90% confidence of 100x bootstraps. The reference genomes indicated as bin X Y are MAGs obtained from the same metagenomes (CSSed10, CSSed11, T3Sed10, T1Sed10, and B1Sed10) for which the recruitment experiment was performed [33]. MAG IDs with an asterisk show the presence of a partial 16S rRNA gene with 94% identity to ‘*Ca.* Syntropholuna alkaliphila’. Presence and absence of genes and pathways are indicated by the symbols on the right hand side. Full = present, empty = partially present. Gene abbreviations for acetate activation: *ack/pta* = acetate kinase/phosphate acetyltransferase, *ACSS/hppA* = acetyl-coA synthetase/H^+^/Na^+^ translocating pyrophosphatases, *paaK* = phenylacetate-CoA ligase. WL-carbonyl and WL-methyl are the carbonyl and methyl branch of the Wood–Ljungdahl pathway, respectively. *PFOR* = pyruvate ferredoxin oxidoreductase. Gene abbreviations encoding for formate dehydrogenases: *fdoGH*, *fdhAB*. Gene abbreviations encoding for [NiFe] hydrogenase: *B-cyt* = *b*-type cytochrome subunit, *hyaBA*.

**FIGURE 6:**
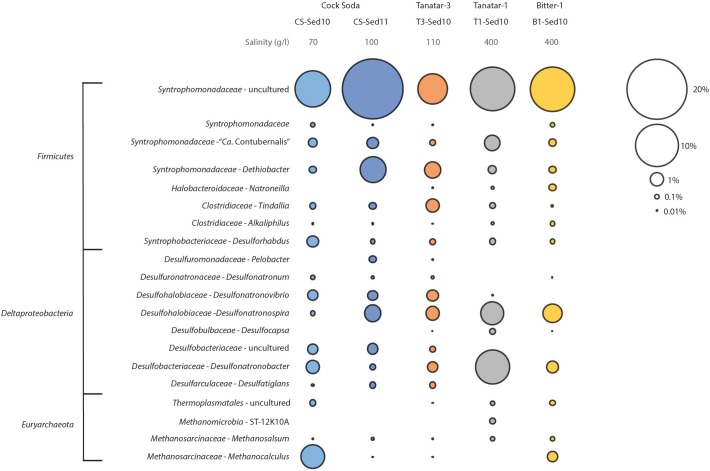
Relative abundance (%) of OTUs of *Firmicutes*, *Deltaproteobacteria*, and *Euryarchaeota* in five different soda lake sediment samples with different salinity. Only reads with higher relative abundance than 0.1% in at least one of the sediment samples are shown.

The SAOB ‘*Ca.* S. acetioxidans’ belongs to the family *Syntrophomonadaceae* (Sorokin et al., 2014a), mostly known for its characterized syntrophic butyrate oxidizers (Müller et al., 2010). Recently, it was shown that members of this family are abundant in hypersaline soda lake sediments from the Kulunda steppe (south-western Siberia) and 52 novel MAGs were retrieved from five metagenomes (Vavourakis et al., 2018). Based on the phylogeny of 16 conserved ribosomal proteins, the ‘*Ca.* S. acetioxidans’ genome derived from our M-SAO culture (MSAO_Bac1) was most closely related to two of those MAGs with similar G+C content, namely *Syntrophomonadaceae* CSSed11_10 (not shown) and T1Sed10_67 (Figure 5). The two MAGs belonged likely to another species (ANI = 96.9%, conDNA = 61%) within the genus ‘*Ca.* Syntrophonatronum’ (ANI = 83.25% with MSAO_Bac1). Since T1Sed10.67 encoded for an AMP-forming acetyl-CoA synthetase and all genes of the WL pathway, it has the genetic potential to perform acetate oxidation, as was the case for ‘*Ca.* S. acetioxidans’ (Figure 5 and Supplementary Data S2). Other candidate (reversed) acetogens able to produce H_2_ in syntrophic interactions based on the predicted genome potential were present in the sediment metagenomes, such as *Dethiobacter alkaliphilus*, CSSed11.298R1 and CSSed11.131 (Figure 5). Some other MAGs belonged to the genus ‘*Ca.* Syntropholuna’ (94% 16S rRNA gene identity; Supplementary Data S3), from which a syntrophic benzoate-degrading culture was recently obtained (Sorokin et al., 2016). This culture was obtained in a study where soda lake enrichment cultures with other fatty acids and alcohols such as butyrate, propionate and ethanol also mainly resulted in syntrophic cultures (Sorokin et al., 2016). Previously obtained 16S rRNA gene profiles of sediments from south-western Siberian hypersaline soda lakes (Vavourakis et al., 2018) were also examined, including lake Bitter-1 from which our enrichment cultures originated (Sorokin et al., 2014a, 2016). *Syntrophomonadaceae* was among the most abundant family of all *Bacteria*; their occurrence ranged from 6.8 to 24.5% at 100 g l^-1^ salinity (Figure 6). The highest identity (up to 99%) with the full 16S rRNA gene sequence of ‘*Ca.* S. acetioxidans’ (Sorokin et al., 2014a) was with representative sequences from the OTU assigned to ‘*Candidatus* Contubernalis’. ‘*Ca.* Contubernalis alkalaceticum’ was the first cultivated syntrophic SAOB obtained from a low-salt soda lake (Zhilina et al., 2005) and ‘*Ca.* S. acetioxidans’ and ‘*Ca.* C. alkalaceticum’ form an independent branch within the family of *Syntrophomonadaceae* (Sorokin et al., 2014a). In line with the metagenomics data, the abundance of this OTU was between 0.2 and 1.4% of all reads in the four examined soda lakes. These combined results show that syntrophic fatty acid oxidation might be an important anaerobic carbon mineralization route in soda lake sediments.

Bacterial 16S rRNA gene community profiling of the same hypersaline soda lake sediment samples showed that the potential acetate-oxidizing SRB clades were present only in very low relative abundance (≤0.1%). Representatives of the genera *Desulfonatronospira* and *Desulfonatronobacter* were the most abundant, followed by *Desulfonatronovibrio* and *Desulfobacteriaceae* sp. (Figure 6). Most cultured representatives of these genera can use formate and H_2_ as electron donor but need or prefer acetate as a carbon source and previous attempts to isolate SRB with acetate as electron donor and sulfate as electron acceptor have failed (Zhilina et al., 1997; Sorokin et al., 2008, 2011, 2012, 2015b; Sorokin and Chernyh, 2017). In fact, the only acetate-oxidizing anaerobes cultivated from soda lakes so far are sulfur-reducing bacteria belonging to the *Chrysiogenetes* and *Halanaerobiales* (Sorokin et al., 2010) and the thiosulfate or sulfite-reducing *Desulfonatronobacter acetoxydans* (Sorokin et al., 2015b).

Archaeal 16S rRNA gene community profiling showed that the most abundant methanogenic clades are methylotrophic and hydrogenotrophic methanogens belonging to *Methanobacterium*, *Methanocalculus*, *Methanolobus*, and *Methanosalsum* (Figure 6). Thermoplasmata were abundant and some MAGs were constructed from this order that belonged to the methyl-reducing methanogens of the order *Methanomassiliicoccales* (Vavourakis et al., 2018). Aceticlastic methanogens from the genera *Methanosaeta* and *Methanosarcina* were present at very low relative abundance (≤0.1%) and each in only one of the soda lake sediment samples. In previous research, aceticlastic methanogenesis did not occur in incubations with several soda lake sediments (Nolla-Ardevol et al., 2012). Since the minimum salinity of the soda lakes investigated here was 70 g l^-1^ (which corresponds to 1.1 M Na^+^ in defined media), our findings are in line with other previous enrichment cultures from several soda lakes that yielded aceticlastic *Methanosaeta* sp. only at salinities below 0.6 M Na^+^, whereas at higher sodium concentrations, syntrophic communities with the extremely salt tolerant hydrogenotrophic methanogenic partner *Methanocalculus* sp. were considered responsible for acetate oxidation (Sorokin et al., 2015a). Since most of the SRB and methanogens in soda lake environments cannot use acetate as electron donor, it seems that acetate would mainly be oxidized by syntrophic associations in soda lake environments with high salinities.

## Conclusion

Based on the results gathered from both the M-SAO and S-SAO cultures, it can be concluded that H_2_ and/or formate are the main electron carriers during SAO since; (1) H_2_ was produced in SAO performing cultures at concentrations that were energetically favorable; (2) inhibition of the sulfate reducing and methanogenic partner resulted in H_2_ accumulation and unfavorable conditions for acetate oxidation; (3) the draft genome of MSAO_Bac1 encoded for intra- and extracellular [NiFe] hydrogenases and formate dehydrogenases and did not encode for formate transporters, but formate and H_2_ were interconverted by the syntrophic partner. The low solubility of H_2_ at soda lake conditions explain why SAO is energetically feasible at the measured H_2_ concentrations. The metagenomic and 16S rRNA gene amplicon data of the five hypersaline soda lake sediment samples, together with previous cultivation efforts, indicated that syntrophy seems to be an important anaerobic process for organic matter degradation at haloalkaline conditions. Most haloalkaliphilic methanogens and SRB do not use acetate for catabolic purposes, which implies that SAO might be the dominant acetate-dependent catabolic process at haloalkaline conditions. Further experiments involving cultivation, labeled substrate addition and metagenomics and metaproteomics will aid to elucidate that this is true for acetate oxidation but also for other fatty acids and alcohols.

## Data Availability

Datasets are in a publicly accessible repository. The raw sequence reads of the metagenomes from the methanogenic SAO enrichment culture have been deposited to the NCBI Sequence Read Archive (SRP156567). The final MAGs described in this paper have been deposited as individual Whole Genome Shotgun projects at DDBJ/EMBL/GenBank (without annotation). Accession numbers are given in Supplementary Data S3 and Supplementary Table S3 (QZAD00000000-QYZW00000000). All versions described in this paper are version XXXX01000000.

## Author Contributions

PT designed the experiments, conducted most of the experimental work, and wrote the article. CV analyzed the metagenomic and 16S rRNA gene amplicon data, and helped with analysis and interpretation of the work and writing of the article. DS provided the cultures and the expertise to grow them, and helped with design, analysis, and interpretation of the experiments. RK performed the thermodynamic calculations, and helped with analysis and interpretation of the work. JD performed the lipid analysis. GM, AS, and CP contributed with experimental design and interpretation of the work. All authors provided feedback and corrections on the manuscript, revised the intellectual content, approved the final version to be published, and agreed to be accountable for all aspects of the work.

## Conflict of Interest Statement

The authors declare that the research was conducted in the absence of any commercial or financial relationships that could be construed as a potential conflict of interest.
