# Supplementary material for: Metabolism and Occurrence of Methanogenic and Sulfate-Reducing Syntrophic Acetate Oxidizing Communities in Haloalkaline Environments
Source: Front Microbiol. 2018 Dec 10;9:3039. doi: 10.3389/fmicb.2018.03039 (PMC6295475; doi:10.3389/fmicb.2018.03039)
Supplement: Supplementary file 1 [file Presentation_1.pdf]

*Supplementary Material*

# Metabolism and occurrence of methanogenic and sulfate-reducing syntrophic acetate oxidizing communities in haloalkaline environments

Peer H.A. Timmers[1,2*], Charlotte D. Vavourakis[3], Robbert Kleerebezem[4], Jaap S. Sinninghe Damsté[5,6], Gerard Muyzer[3], Alfons J.M. Stams[1,7], Dimity Y. Sorokin[4,8], Caroline M. Plugge[1,2]

[1]Laboratory of Microbiology, Wageningen University & Research, Stippeneng 4, 6708 WE Wageningen, The Netherlands

[2]Wetsus, European Centre of Excellence for Sustainable Water Technology, Oostergoweg 9, 8911 MA Leeuwarden, The Netherlands

[3]Department of Freshwater and Marine Ecology, Institute for Biodiversity and Ecosystem Dynamics, University of Amsterdam, The Netherlands

[4]Department of Biotechnology, Delft University of Technology, The Netherlands

[5]NIOZ Netherlands Institute for Sea Research, Department of Marine Microbiology and Biogeochemistry and Utrecht University, PO Box 59, 1790 AB Den Burg, The Netherlands

[6]Faculty of Geosciences, Department of Earth Sciences, Utrecht University, 3508 TA Utrecht, The Netherlands

[7]Centre of Biological Engineering, University of Minho, Campus de Gualtar, 4710-057, Braga, Portugal

[8]Winogradsky Institute of Microbiology, Research Centre of Biotechnology, Russian Academy of Sciences, Leninskii avenue 14, Moscow, Russia

***Correspondence:**

Dr Peer HA Timmers

peer.timmers@kwrwater.nl

## 1. Supplementary discussion

### 1.1 Stoichiometry of acetate oxidation

In the M-SAO and S-SAO cultures, around 4-5 mM and 1.6 mM acetate was consumed that did not result in stoichiometric methane and sulfide formation, respectively. *M. natronophilus* needs acetate for growth (Zhilina et al., 2013), whereas *Desulfonatronovibrio magnus* does not need it but grows better with it (Sorokin et al., 2011). Pure growing cultures of *M. natronophilus* consumed on average 1.1 mM (±0.5) acetate and pure cultures of *Desulfonatronovibrio magnus* consumed on average 0.6 mM (±0.4) acetate when growing with $H_2$ as electron donor (Supplementary Fig 11). Anabolic acetate consumption can therefore explain the gap in acetate stoichiometry, since a total of 8 MAGs were recovered from the enrichment culture that represent 8 organisms that could have consumed part of the acetate for growth (Supplementary Table 2, Supplementary Fig 1). Closely related cultured representatives of the recovered MAGs that can use acetate as carbon source are *Methanocalculus natronophilus* AMF5 (MSAO_Arc1 and MSAO_Arc2), *Tindallia magadiensis* strain Z-7934 (MSAO_Bac2), *Desulfonatronospira thiodismutans* (MSAO_Bac3), and *Desulfonatronovibrio hydrogenovorans* (MSAO_Bac4) (Kevbrin et al., 1998;Sorokin et al., 2008;Zhilina et al., 2013;Sorokin et al., 2015). All bacterial and archaeal MAGs contained genes for acetate activation either via ACK/PTA (MSAO_Bac2, MSAO_Bac3) or via AMP-forming acetyl-CoA synthethase (MSAO_Bac1, MSAO_Bac4 and all archaeal MAGs). Besides '*Ca.* S. acetioxidans', only MSAO_Bac3 (related to *Desulfonatronospira* sp.) contained all genes for operating the Wood-Ljungdahl (WL) pathway, but pure culture representatives of this genus did not use acetate for catabolic purposes (Zhilina et al., 1997;Sorokin et al., 2008;Sorokin et al., 2010) and it therefore probably uses the WL pathway for $CO_2$ fixation. The other archaeal MAGs were a lithotrophic methanogen (MSAO_Arc2) and a methylotrophic methanogen (MSAO_Arc3), both incapable to use acetate for methanogenesis.

### 1.2 Other metabolic properties

The genome of '*Ca.* S. acetioxidans' encodes all enzymes of the glycolysis (except for the pyruvate kinase isozymes) which shows that it probably has the ability to degrade sugars or to perform gluconeogenesis The genome also encodes for the non-oxidative branch of the pentose phosphate pathway and can therefore produce glyceraldehyde-3-phosphate from ribulose-5-

phosphate and vice versa (Supplementary Fig 7). '*Ca.* S. acetioxidans' does not encode for a complete TCA cycle. Conversion of malate to oxaloacetate proceeds via activity of a malate dehydrogenase, which is not present in the genome. However, the genome does encode for an enzyme that could bypass this conversion by producing pyruvate from malate using a $NAD^+$-dependent oxaloacetate-decarboxylating malate dehydrogenase (ME2; k121-4746-cds5). The pyruvate could also come from acetate via activity of pyruvate synthase (k121-561). Pyruvate could also be produced from oxaloacetate via oxaloacetate decarboxylase (k121-5682) (Supplementary Fig 7). '*Ca.* S. acetioxidans' does not encode for the full enzyme of pyruvate carboxylase since it only encodes for subunit B and could therefore not produce oxaloacetate from pyruvate. It therefore probably has to proceed via PEP. The enzyme that normally goes in the direction from PEP to pyruvate, pyruvate kinase is encoded in the genome (k121-951). The other missing part of the TCA cycle is a gene that encodes for the enzyme for conversion of citrate to isocitrate via cis-aonitate; aconitate hydratase (acnA/acnB/ACO). The genome does not encode for an aconitase. The genome does encode for a possible homologue of aconitase, the 3-isopropylmalate dehydratase (k121-5682). 3-isopropylmalate dehydratase (alpha-isopropylmalate isomerase) was described to be a possible aconitase homologue that also belongs to the aconitase superfamily where all members show a similar overall structure and domain organization. 3-isopropylmalate dehydratase is normally involved in leucine biosynthesis. The gene for 3-isopropylmalate dehydratase was indeed found next to oxaloacetate decarboxylase and leucine biosynthesis genes (k121-5682) and is therefore probably involved in leucine biosynthesis and not in aconitase activity. This however needs to be proven. '*Ca.* S. acetioxidans' therefore also probably does not have the potential to fix carbon using the reverse TCA cycle, even with the recently discovered reversibility of citrate synthase (Mall et al., 2018;Nunoura et al., 2018), since the genome also does not encode for a pyruvate carboxylase gene.

## 2. Supplementary Figures and Tables

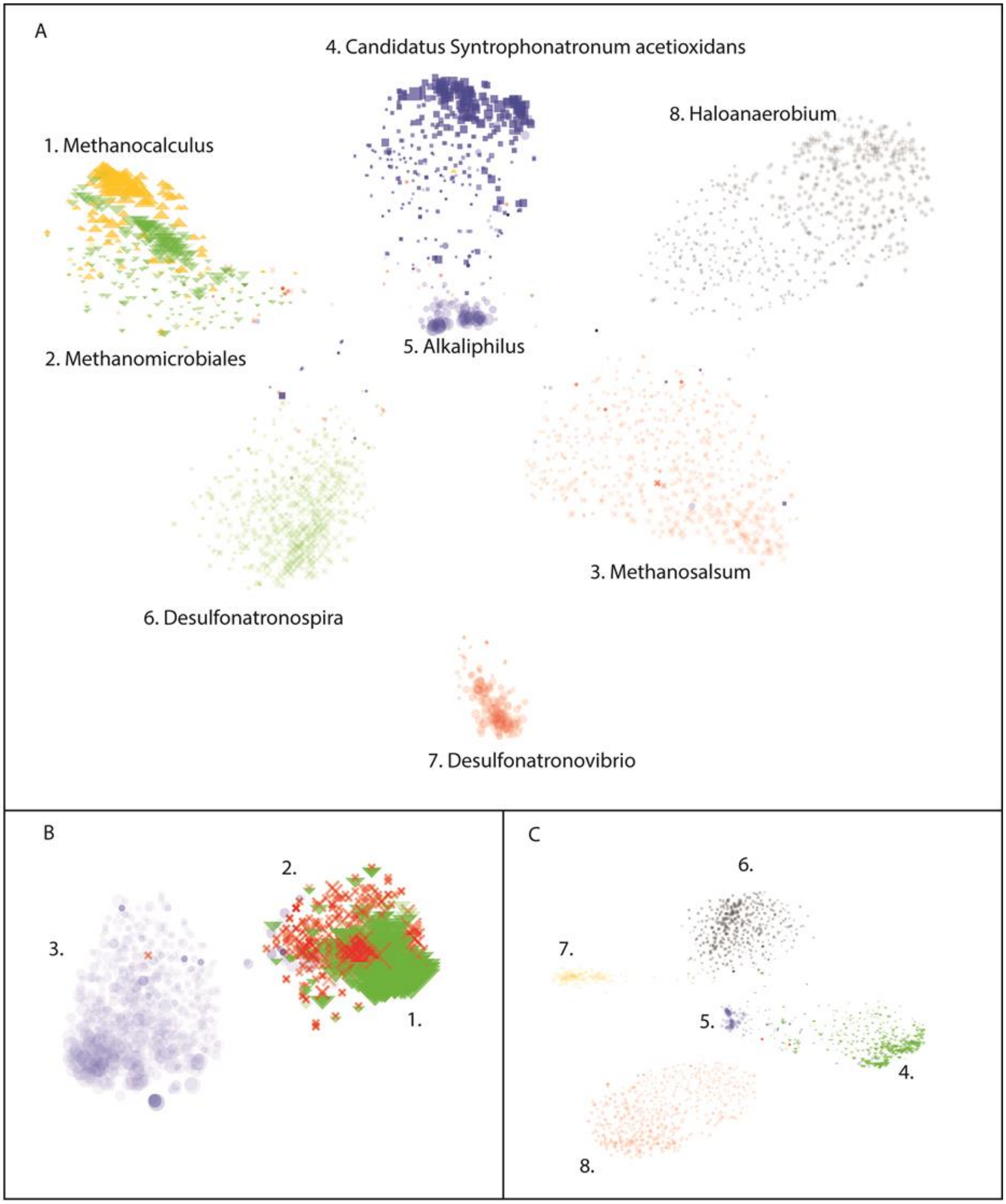


**Figure S1** Principal component analysis plot (Vizbin) showing the 8 MAGs reconstructed from the methanogenic enrichment culture performing syntrophic acetate oxidation (M-SAO). A) All bins, B) only Archaeal bins, C) Bacterial bins. 1. MSAO_Arc1, 2. MSAO_Arc2, 3. MSAO_Arc3, 4. MSAO_Bac1, 5. MSAO_Bac2, 6. MSAO_Bac3, 7. MSAO_Bac4, 8. MSAO_Bac5.

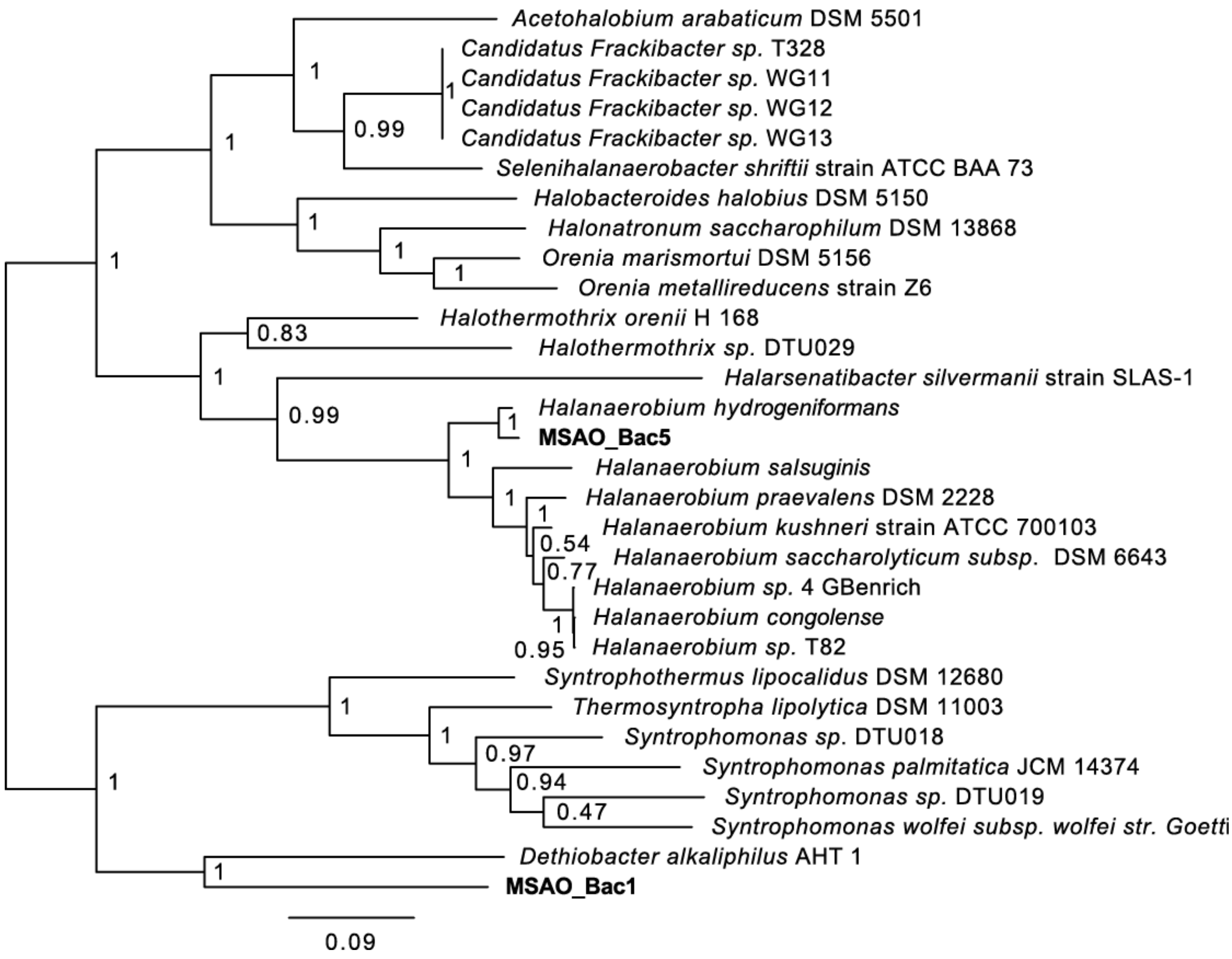


**Figure S2** Maximum likelihood phylogenetic tree of a set of 16 ribosomal proteins confirming the affiliation of MSAO_Bac1 with the family *Syntrophomonadaceae* and of MSAO_Bac5 with the genus *Halanaerobium* within the family *Halanaerobiaceae*. Node values show the fraction of 100x bootstraps values.

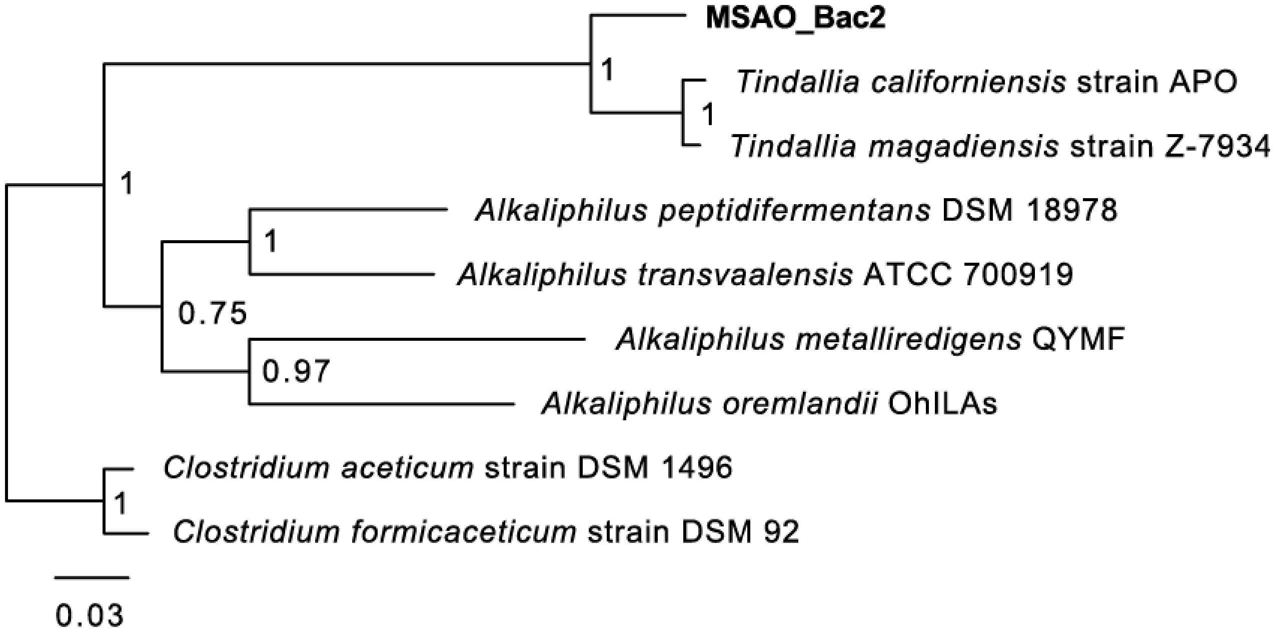


**Figure S3** Maximum likelihood phylogenetic tree of a set of 16 ribosomal proteins confirming the affiliation of MSAO_Bac2 with the genus *Tindallia* within the family *Clostridiaceae*. Node values show the fraction of 100x bootstraps values.

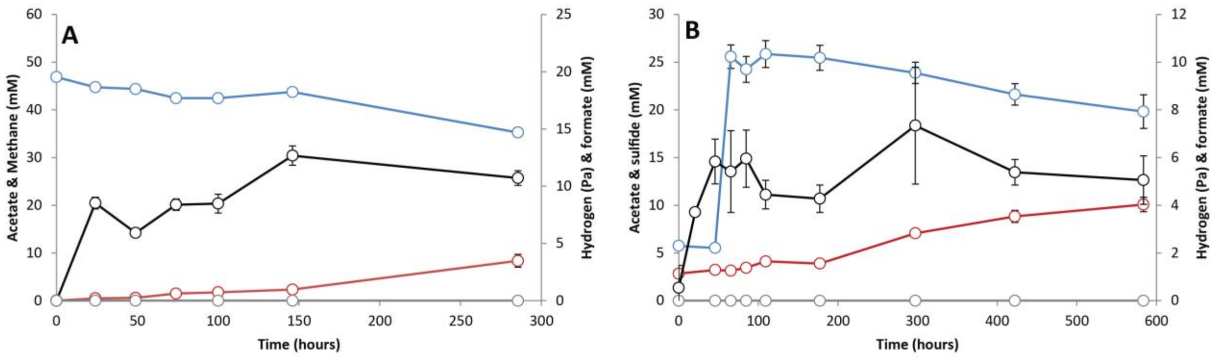


**Figure S4** Activity test results with pre-grown syntrophic acetate oxidizing cultures with A) a methanogenic partner (M-SAO) or B) a sulfate-reducing partner (S-SAO). Lines represent acetate (blue line), $H_2$ (black line), formate (grey line) and methane or sulfide (red line) evolution in cultures supplemented with acetate and shaken at 130 rpm.

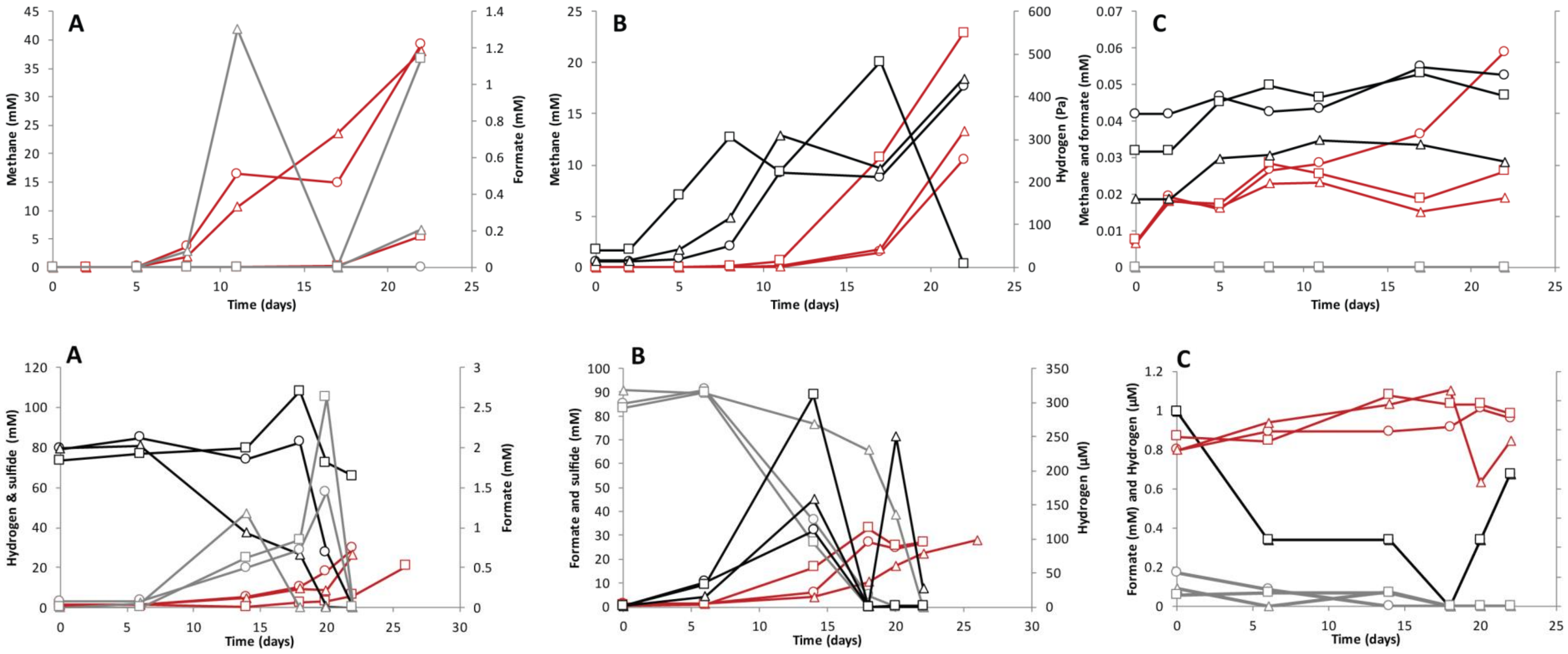


**Figure S5** Pure cultures of *Methanocalculus natronophilus* strain AMF5 (top graphs) and *Desulfonatronovibrio magnus* (bottom graphs) growing on A) 100% hydrogen, B) 100 mM formate, and C) no electron donor with acetate as carbon source – all shaken at 130 rpm, showing $H_2$ (black line), formate (grey line) and methane or sulfide (red line) evolution. Each line represents a biological replicate incubation.

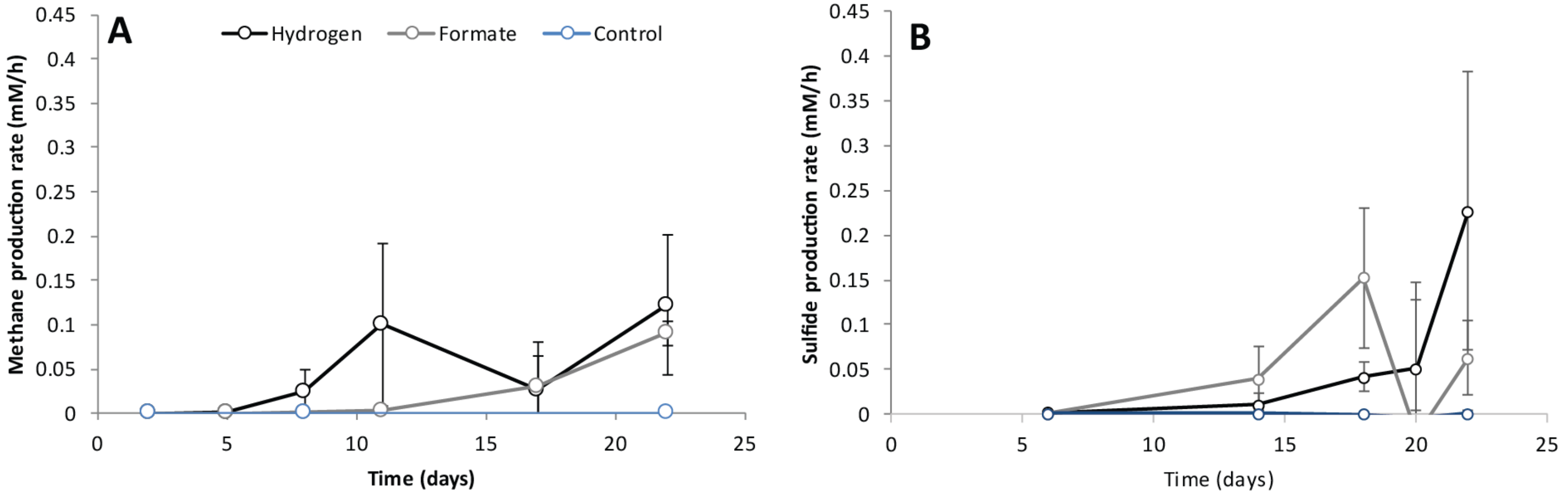


**Figure S6** Methane (A) and sulfide (B) production rates (mM $h^{-1}$) of pure cultures of *Methanocalculus natronophilus* strain AMF5 and *Desulfonatronovibrio magnus*, respectively. Pure cultures were either growing on 100% $H_2$ (black lines), 100 mM formate (grey lines), or no electron donor (blue lines) – all shaken at 130 rpm. Standard deviations represent biological triplicate incubations.

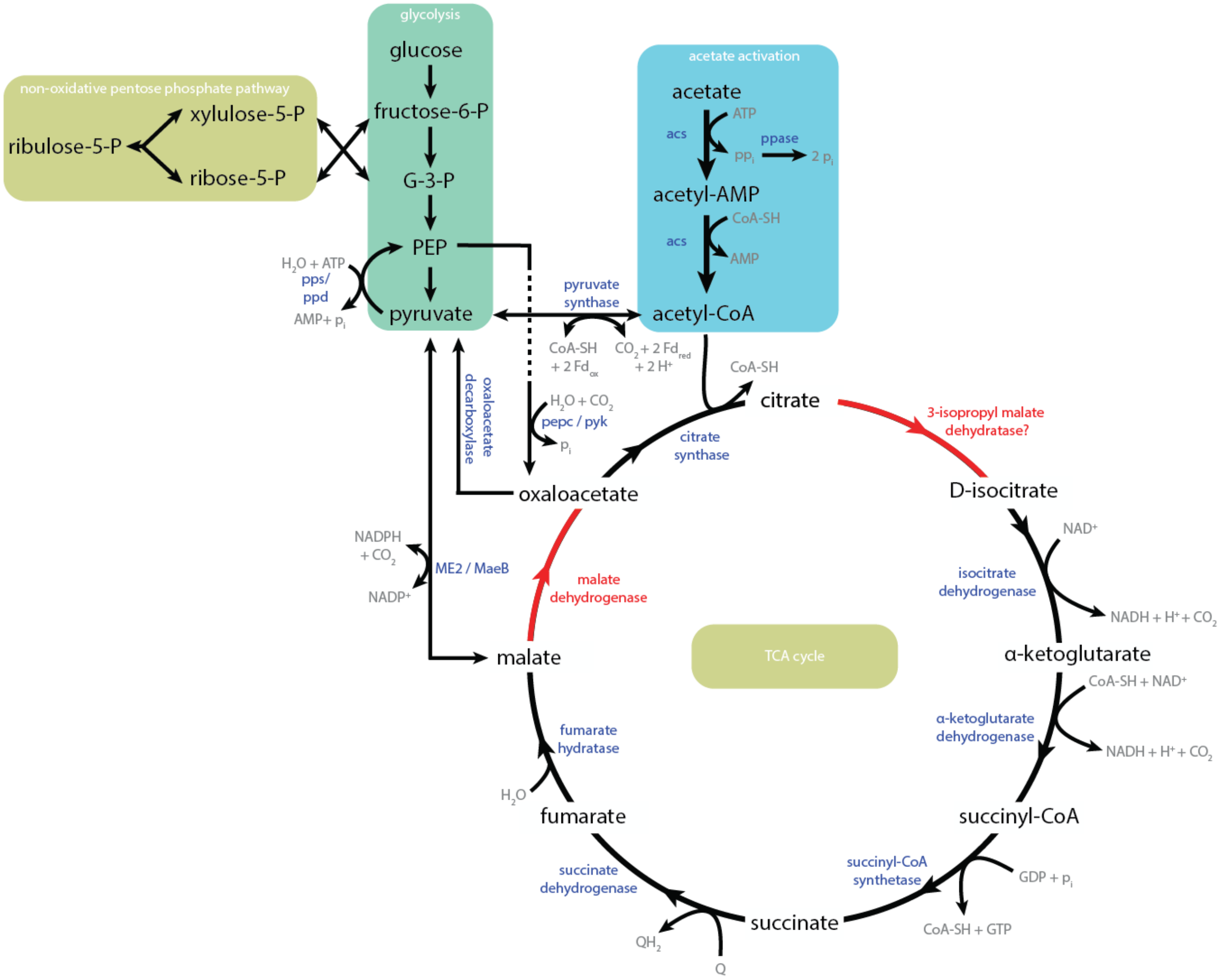


**Figure S7** Schematic reconstruction of acetate activation, the TCA cycle, the non-oxidative pentose phosphate pathway and glycolysis/gluconeogenesis in the partial genome of '*Ca.* Syntrophonatronum acetixodidans'. Red names and red lines indicate absence of these genes or these conversions, respectively.

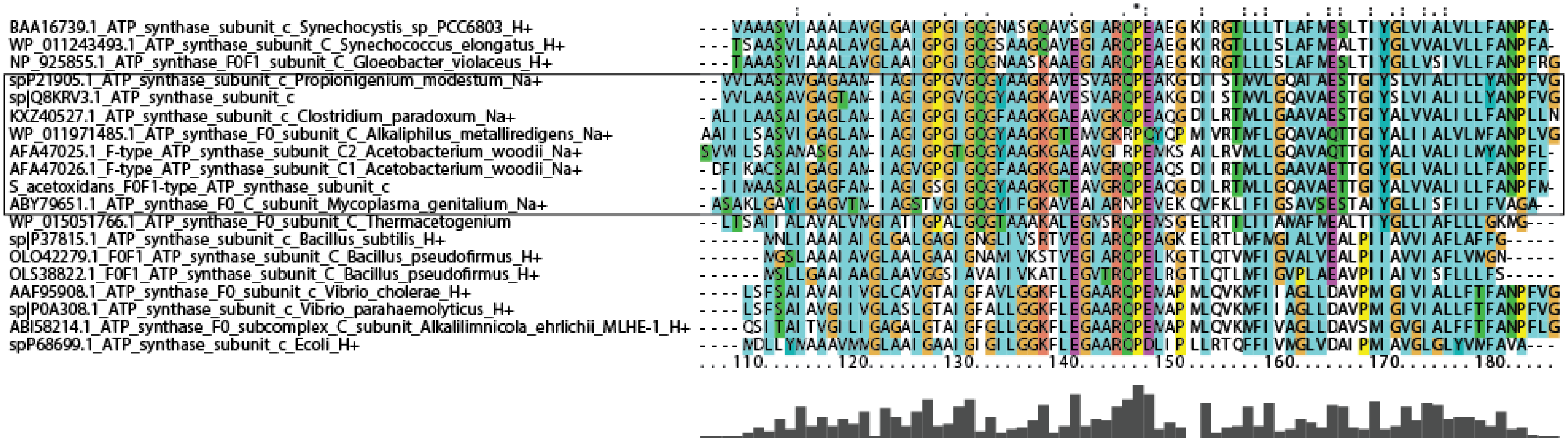


**Figure S8** Partial sequence alignments of sodium or proton-dependent $F_1F_0$-type ATP synthase c-subunits with the one of '*Ca.* Syntrophonatronum acetixodidans'. Boxed sequences show sodium dependent $F_1F_0$-type ATP synthase c-subunits whereas all others are proton-dependent. *Thermoacetogenium phaeum* has both amino acids found in sodium- and proton dependent $F_1F_0$-type ATP synthase c-subunits.

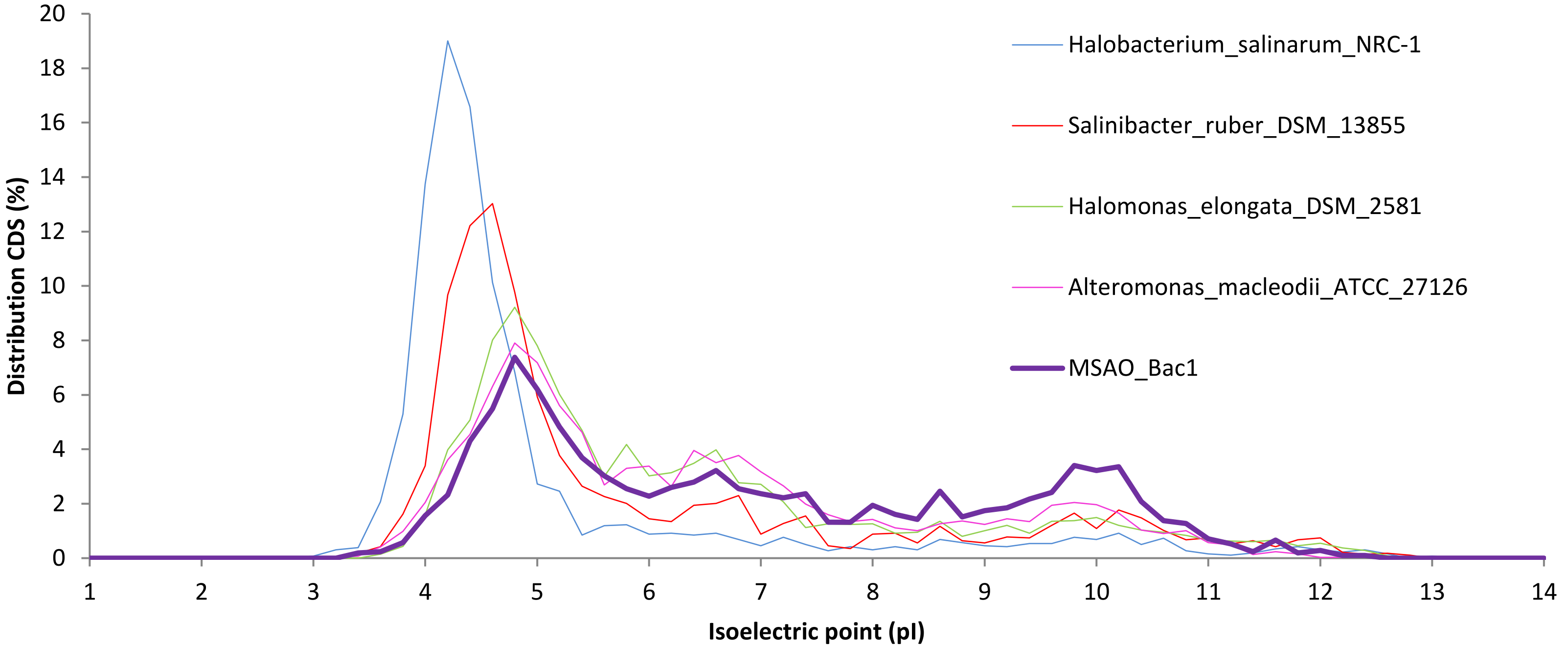


**Figure S9** Isolelectric points (pI) of the predicted proteome of '*Ca.* Syntrophonatronum acetixodidans' as compared to other microorganisms with acidic proteomes: halophilic microorganisms with highly acidic proteomes that use the salt-in strategy (*Halobacterium* and *Salinibacter*), the moderate halophilic aerobe *Halomonas elongate* that accumulates organic osmotic solutes, and the aerobic non-halophilic marine *Alteromonas maclodii*.

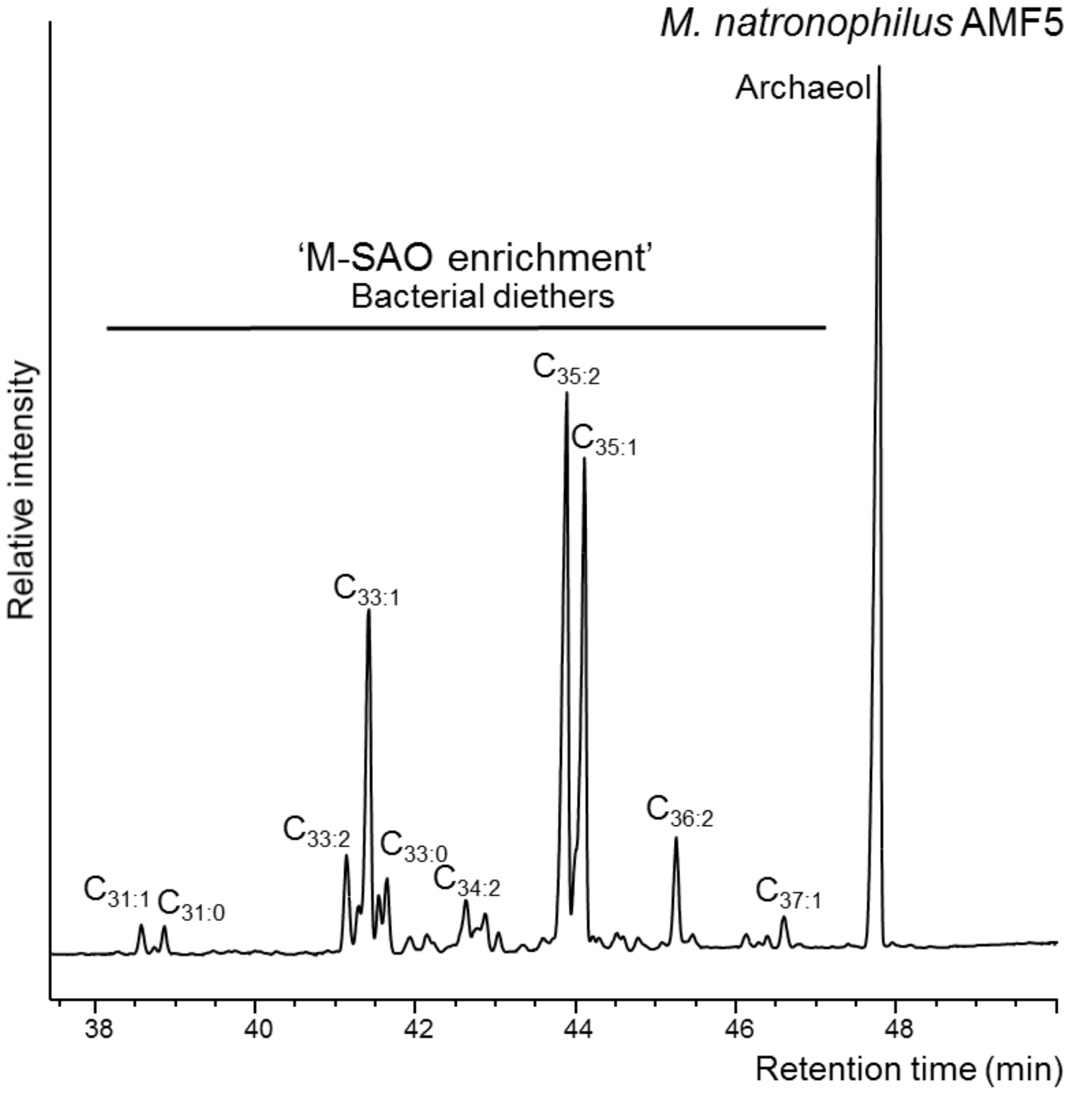


**Figure S10** Partial gas chromatogram showing the predominant lipids of the M-SAO enrichment culture and the pure culture methanogen *M. natronophilus* AMF5 after acid hydrolysis of the Bligh-Dyer extract of freeze dried cell material.

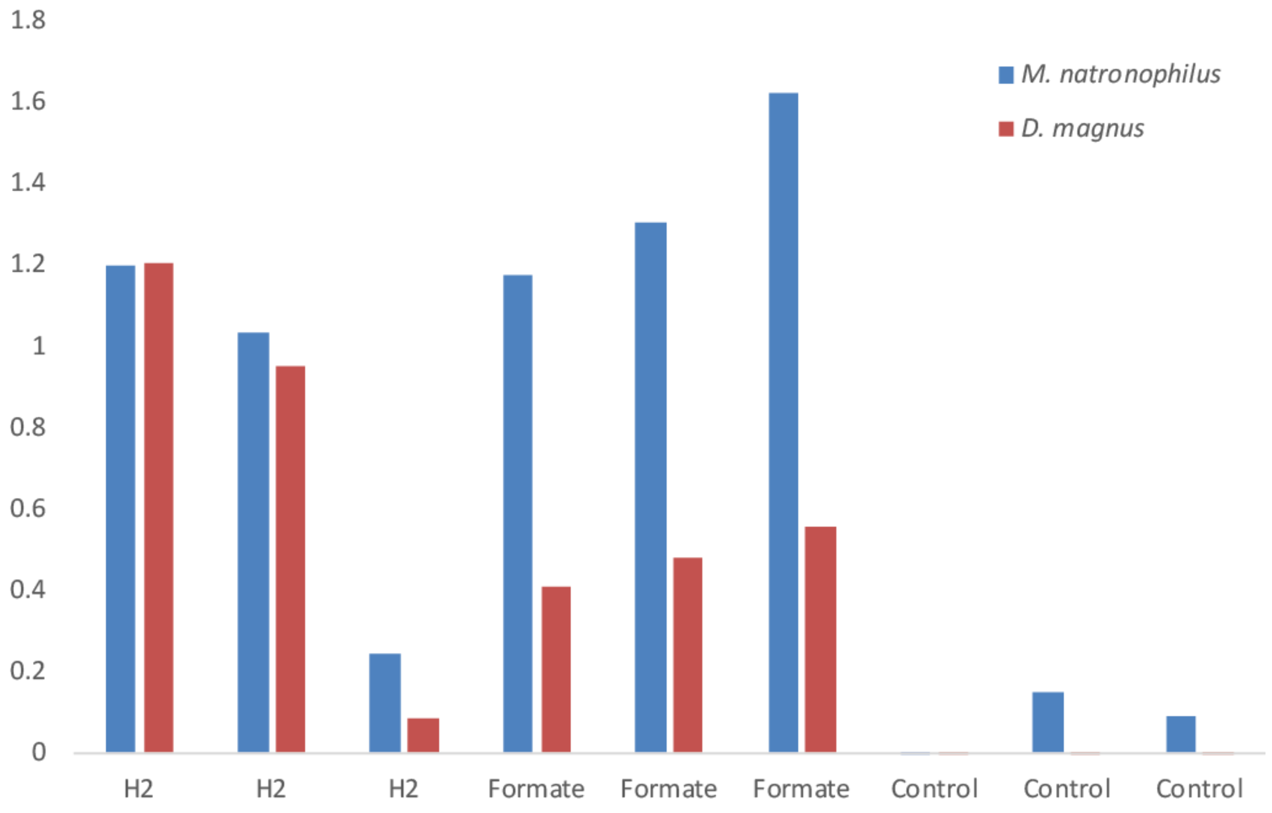


**Figure S11** Pure cultures of *Methanocalculus natronophilus* strain AMF5 (red bars) and *Desulfonatronovibrio magnus* (blue bars) growing on 100% $H_2$, 100 mM formate, or no electron donor (control) with acetate as carbon source – all shaken at 130 rpm, showing anabolic acetate consumption during 22 days of incubation. Each line represents a biological replicate incubation.

**Table S1** Genome characteristics of the final eight MAGs from the organisms present with reasonable abundance in the methanogenic SAO enrichment culture. Completeness and contamination estimates were calculated using CheckM, sequence coverage estimates were obtained by mapping the raw reads with Bowtie2.

| MAG ID | Estimated completeness (%) | Estimated contamination (%) | G+C mol% | # Contigs | Length (Mb) | #CDS | Coding density (%) | #tRNA | #5S rRNA | #16S rRNA | #23S rRNA | Estimated coverage (x) |
|---|---|---|---|---|---|---|---|---|---|---|---|---|
| MSAO_Arc1 | 100 | 0 | 52.7 | 127 | 1.78 | 1,849 | 92.4 | 35 | 0 | 1 | 0 | 931 |
| MSAO_Arc2 | 97 | 1 | 50.5 | 232 | 1.69 | 1,757 | 92.5 | 37 | 1 | 0 | 0 | 235 |
| MSAO_Arc3 | 87 | 2 | 34.7 | 594 | 1.53 | 1,467 | 92.9 | 30 | 2 | 1 | 0 | 11 |
| MSAO_Bac1 | 90 | 12 | 44.3 | 300 | 1.97 | 1,876 | 91.7 | 39 | 1 | 1 | 1 | 665 |
| MSAO_Bac2 | 97 | 7 | 42.3 | 129 | 2.48 | 2,271 | 92.0 | 38 | 2 | 0 | 0 | 43 |
| MSAO_Bac3 | 99 | 2 | 53.1 | 391 | 2.90 | 2,632 | 89.1 | 40 | 0 | 1 | 0 | 22 |
| MSAO_Bac4 | 86 | 17 | 43.9 | 133 | 2.28 | 1,998 | 89.3 | 22 | 0 | 0 | 0 | 20 |
| MSAO_Bac5 | 87 | 2 | 33.4 | 566 | 2.03 | 1,807 | 94.7 | 37 | 2 | 0 | 0 | 17 |

**Table S2** Taxonomic assignments of the eight final MAGs presented in this study, based on 16S rRNA gene (*manually placed in correct bin) and gene contig annotations against NCBI-nr.

| MAG ID | CheckM lineage | 16S rRNA gene besthit (% identity, e-value) | Gene contigs besthit (% total hits) |
|---|---|---|---|
| MSAO_Arc1 | p_Euryarchaeota UID54 | *Methanocalculus* sp. AMF5 (100;0)* | *Methanofollis liminatans* (16) |
| MSAO_Arc2 | p_Euryarchaeota UID54 | NA | *Methanofollis liminatans* (15) |
| MSAO_Arc3 | p_Euryarchaeota UID49 | *Methanosalsum natronophilum* strain AME2 (99;0)* | *Methanosalsum zhilinae* (77) |
| MSAO_Bac1 | p_Firmicutes UID241 | "*Ca.* Syntrophonatronum acetioxidans clone AAS1" (100;0)* | *Dethiobacter alkaliphilus* (25) |
| MSAO_Bac2 | o_Clostridiales UID1120 | *Alkaliphilus metalliredigens* QYMF (93;2e-92)*, *Tindallia magadiensis* strain Z-7934 (97;2e-91)* | *Alkaliphilus metalliredigens* (19) |
| MSAO_Bac3 | c_Deltaproteobacteria UID3217 | *Desulfonatronospira* sp. AHT34 (99;0) | *Desulfonatronospira thiodismutans* (94) |
| MSAO_Bac4 | c_Deltaproteobacteria UID3217 | *Desulfonatronovibrio* sp. (99;0)* | *Desulfonatronovibrio hydrogenovorans* (77) |
| MSAO_Bac5 | p_Firmicutes UID241 | *Halanaerobium acetoethylicum* (99;3e-62) | *Halanaerobium hydrogeniforans* (89) |

**Table S3** Organism names and WGS accession numbers (NCBI) of the eight MAGs described in this paper

| MAG ID | Organism name | WGS accession |
|---|---|---|
| MSAO_Arc1 | *Methanocalculus* sp. MSAO_Arc1 | QZAD00000000 |
| MSAO_Arc2 | *Methanocalculus* sp. MSAO_Arc2 | QZAC00000000 |
| MSAO_Arc3 | *Methanosalsum* sp. MSAO_Arc3 | QZAB00000000 |
| MSAO_Bac1 | "*Candidatus* Syntrophonatronum acetioxidans MSAO_Bac1" | QZAA00000000 |
| MSAO_Bac2 | *Tindallia* sp. MSAO_Bac2 | QYZZ00000000 |
| MSAO_Bac3 | *Desulfonatronospira* sp. MSAO_Bac3 | QYZY00000000 |
| MSAO_Bac4 | *Desulfonatronovibrio* sp. MSAO_Bac4 | QYZX00000000 |
| MSAO_Bac5 | *Halanaerobium* sp. MSAO_Bac5 | QYZW00000000 |

## References


Kevbrin, V.V., Zhilina, T.N., Rainey, F.A., and Zavarzin, G.A. (1998). Tindallia magadii gen. nov., sp. nov.: An alkaliphilic anaerobic ammonifier from soda lake deposits. *Current Microbiology* 37**,** 94-100.

Mall, A., Sobotta, J., Huber, C., Tschirner, C., Kowarschik, S., Bacnik, K., Mergelsberg, M., Boll, M., Hugler, M., Eisenreich, W., and Berg, I.A. (2018). Reversibility of citrate synthase allows autotrophic growth of a thermophilic bacterium. *Science* 359**,** 563-567.

Nunoura, T., Chikaraishi, Y., Izaki, R., Suwa, T., Sato, T., Harada, T., Mori, K., Kato, Y., Miyazaki, M., Shimamura, S., Yanagawa, K., Shuto, A., Ohkouchi, N., Fujita, N., Takaki, Y., Atomi, H., and Takai, K. (2018). A primordial and reversible TCA cycle in a facultatively chemolithoautotrophic thermophile. *Science* 359**,** 559-563.

Sorokin, D.Y., Abbas, B., Merkel, A.Y., Rijpstra, W.I.C., Damste, J.S.S., Sukhacheva, M.V., and Van Loosdrecht, M.C.M. (2015). Methanosalsum natronophilum sp nov., and Methanocalculus alkaliphilus sp nov., haloalkaliphilic methanogens from hypersaline soda lakes. *International Journal of Systematic and Evolutionary Microbiology* 65**,** 3739-3745.

Sorokin, D.Y., Rusanov, Ii, Pimenov, N.V., Tourova, T.P., Abbas, B., and Muyzer, G. (2010). Sulfidogenesis under extremely haloalkaline conditions in soda lakes of Kulunda Steppe (Altai, Russia). *Fems Microbiology Ecology* 73**,** 278-290.

Sorokin, D.Y., Tourova, T.P., Henstra, A.M., Stams, A.J., Galinski, E.A., and Muyzer, G. (2008). Sulfidogenesis under extremely haloalkaline conditions by Desulfonatronospira thiodismutans gen. nov., sp. nov., and Desulfonatronospira delicata sp. nov. - a novel lineage of Deltaproteobacteria from hypersaline soda lakes. *Microbiology* 154**,** 1444-1453.

Sorokin, D.Y., Tourova, T.P., Kolganova, T.V., Detkova, E.N., Galinski, E.A., and Muyzer, G. (2011). Culturable diversity of lithotrophic haloalkaliphilic sulfate-reducing bacteria in soda lakes and the description of Desulfonatronum thioautotrophicum sp. nov., Desulfonatronum thiosulfatophilum sp. nov., Desulfonatronovibrio thiodismutans sp. nov., and Desulfonatronovibrio magnus sp. nov. *Extremophiles* 15**,** 391-401.

Zhilina, T.N., Zavarzin, G.A., Rainey, F.A., Pikuta, E.N., Osipov, G.A., and Kostrikina, N.A. (1997). Desulfonatronovibrio hydrogenovorans gen. nov., sp. nov., an alkaliphilic, sulfate-reducing bacterium. *Int J Syst Bacteriol* 47**,** 144-149.

Zhilina, T.N., Zavarzina, D.G., Kevbrin, V.V., and Kolganov, T.V. (2013). [Methanocalculus natronophilus sp. nov., a new alkaliphilic hydrogenotrophic methanogenic archaeon from a soda lake, and proposal of the new family Methanocalculaceae]. *Mikrobiologiia* 82**,** 681-690.
